# cTAGE5/MEA6 Regulates LBR Localization to Maintain Nuclear Envelope Integrity and Safeguard Against Aging

**DOI:** 10.1111/acel.70185

**Published:** 2025-07-30

**Authors:** Yaqing Wang, Pengyu Sun, Fuqiang Yang, Tiantian Ma, Junwan Fan, Lei Shi, Nan Li, Fei Gao, Kangmin He, Zhiheng Xu

**Affiliations:** ^1^ State Key Laboratory of Molecular Developmental Biology Institute of Genetics and Developmental Biology, Chinese Academy of Sciences Beijing China; ^2^ University of Chinese Academy of Sciences Beijing China; ^3^ State Key Laboratory of Reproductive Biology Institute of Zoology, Chinese Academy of Sciences Beijing China

**Keywords:** aging, cTAGE5/MEA6, LBR, nuclear envelope, senescence

## Abstract

cTAGE5/MEA6 plays a pivotal role in COPII complex assembly, ER‐to‐Golgi trafficking, and secretion. However, whether cTAGE5/MEA6 is involved in other cellular functions remains unclear. Here, we show that conditional *cTAGE5* knockout results in embryonic lethality during development and premature aging in adult mice. *cTAGE5* deficiency leads to abnormal nuclear structure and disturbed cell proliferation in MEF cells. Further mechanistic studies reveal that cTAGE5 localizes not only to the ER exit sites but also to other ER structures, where it interacts with the lamin B receptor (LBR). Loss of cTAGE5 disrupts LBR's localization to the inner nuclear membrane, leading to its retention in the ER and instability. This results in abnormal nuclear (envelope) morphology and cellular senescence, likely driven by activation of the P53/P21 senescence pathway. Thus, our study uncovers cTAGE5's role in maintaining nuclear envelope integrity and highlights its function and potential mechanism in preventing cellular senescence and animal aging.

## Graphical abstract

cTAGE5 regulates LBR trafficking between the ER and the nucleus membrane to maintain the integrity of both the ER and the nuclear envelope. cTAGE5 KO results in LBR retention in the ER, reduced stability, and subsequent disruption of nuclear envelope integrity.

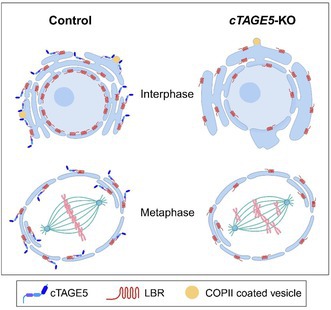

## 1 Introduction

Cutaneous T‐cell lymphoma‐associated antigen 5 (cTAGE5), also known as meningioma‐expressed antigen 6 (MEA6), is upregulated in various tumor tissues and cell lines (Heckel et al. 1997; Comtesse et al. 2002; Kalnina et al. 2008). cTAGE5 has been shown to localize to the endoplasmic reticulum (ER) exit sites (ERES) and interact with TANGO1 and SEC12, participating in the regulation of collagen secretion (Saito et al. 2011, 2014). Importantly, cTAGE5 is indispensable for the initial assembly of the COPII complex, regulating the ER‐to‐Golgi trafficking and secretion of VLDL in the liver, proinsulin in the pancreas, and PTN secretion in oligodendrocytes as well as cellular component transport in neurons (Wang et al. 2016; Fan et al. 2017; Ma et al. 2024; Zhang et al. 2018). *cTAGE5* deficiency leads to the dilation of ER, likely due to ER stress caused by a defect in ER‐to‐Golgi trafficking (Ma et al. 2024). Although the role of cTAGE5 in ER‐to‐Golgi trafficking in cells and several organs has been well studied, its other cellular functions and its role in mammalian development and survival remain unexplored.

The nuclear envelope consists of two parallel but discontinuous membranes. Nuclear membrane proteins, nuclear pore complexes, and nuclear lamins all play essential roles in maintaining nuclear envelope integrity and cell survival (Dey and Baum 2021; Liu et al. 2018; Moir et al. 2000). The inner nuclear membrane is smooth on its surface but features a dense fibrous network known as the nuclear lamina on its inner surface. Over 100 proteins have been identified on the inner nuclear membrane, among which the most well‐known is the lamin B receptor (LBR) (Lukásová et al. 2018). LBR interacts with lamin proteins and is responsible for the integrity of the nuclear envelope (Nikolakaki et al. 2017; Lee et al. 2021; Lukásová et al. 2017). Mutations of both LBR and lamin proteins cause a wide spectrum of human genetic diseases with phenotypes including muscular dystrophy, lipodystrophy, cardiomyopathy, and the premature aging disorders (Nikolakaki et al. 2017; Sánchez‐López et al. 2021). Cells lacking LBR or lamin B1 exhibit nuclear deformation, abnormal cell proliferation, increased chromosome numbers, and micronuclei, resulting in genomic instability and cellular senescence (Lukásová et al. 2018; Bin Imtiaz et al. 2021). LBR is synthesized in the ER and is very likely to reach the nuclear envelope membrane through lateral diffusion (Ungricht et al. 2015). However, whether any specific regulatory factors are involved is not clear.

The outer nuclear membrane is rough, often has ribosomal particles typically attached, and is usually continuous with the rough ER (rER), connecting the perinuclear space to the ER lumen (Güttinger et al. 2009). Thus, the outer nuclear membrane can be considered a specialized region of the rER. During cell division, the ER also undergoes disassembly and reconstruction just like the nucleus (Schwarz and Blower 2016). The transformation of tubular ER into flattened ER sheets is a rate‐limiting step in nuclear envelope remodeling and expansion during the cell cycle (Schwarz and Blower 2016). While the deformation and proper expansion of the ER are believed to be important for nuclear envelope remodeling (Merta et al. 2021), the role of ER proteins and the underlying mechanism in maintaining nuclear envelope integrity remain largely unclear.

Here, we find that cTAGE5 is localized not only at the ER exit sites shown previously but also on other ER structures. We demonstrate that cTAGE5 is crucial for nuclear envelope integrity by maintaining LBR stability and its distribution in the inner nuclear membrane. Knockout of *cTAGE5* in mouse embryonic fibroblast (MEF) cells results in abnormal nuclear morphology and cellular senescence, while its deletion leads to embryonic lethality during development and premature aging in adult mice.

## 2 Results

### 2.1 Knockout of *cTAGE5* Leads to Embryonic Lethality and Defects in Nuclear Morphology and Cell Proliferation in MEF Cells

*cTAGE5* ^*FL/FL*^ mice, generated previously (Wang et al. 2016), were crossed with *Zp3‐Cre* mice to get *cTAGE5* ^*Δ/+*^. Mating of *cTAGE5* ^*Δ/+*^ mice was expected to produce homozygous *cTAGE5* ^*Δ/Δ*^ offspring; however, no homozygous mice were detected among 120 newborns. Dissection of pregnant mice revealed Mendelian ratios of homozygous embryos at embryonic day E8.5, but at E9.5, the homozygous embryos were atrophied, indicating that *cTAGE5* ^*Δ/Δ*^ homozygous embryos die between E8.5 and E9.5 (Table 1). This suggests that cTAGE5 is essential for cell fate determination and embryonic development.

**TABLE 1:** Genotyping of embryos dissected from the pregnant mice.

Stage	*cTAGE5* ^+/+^	*cTAGE5* ^Δ/+^	*cTAGE5* ^Δ/Δ^
E7.5	6	11	7 (alive)
E8.5	6	10	5 (alive)
E9.5	12	20	4 (small, dead)
E12.5	8	13	0
P1	10	19	0

Abbreviations: E, embryonic day; P, postnatal day.

**FIGURE 1:**
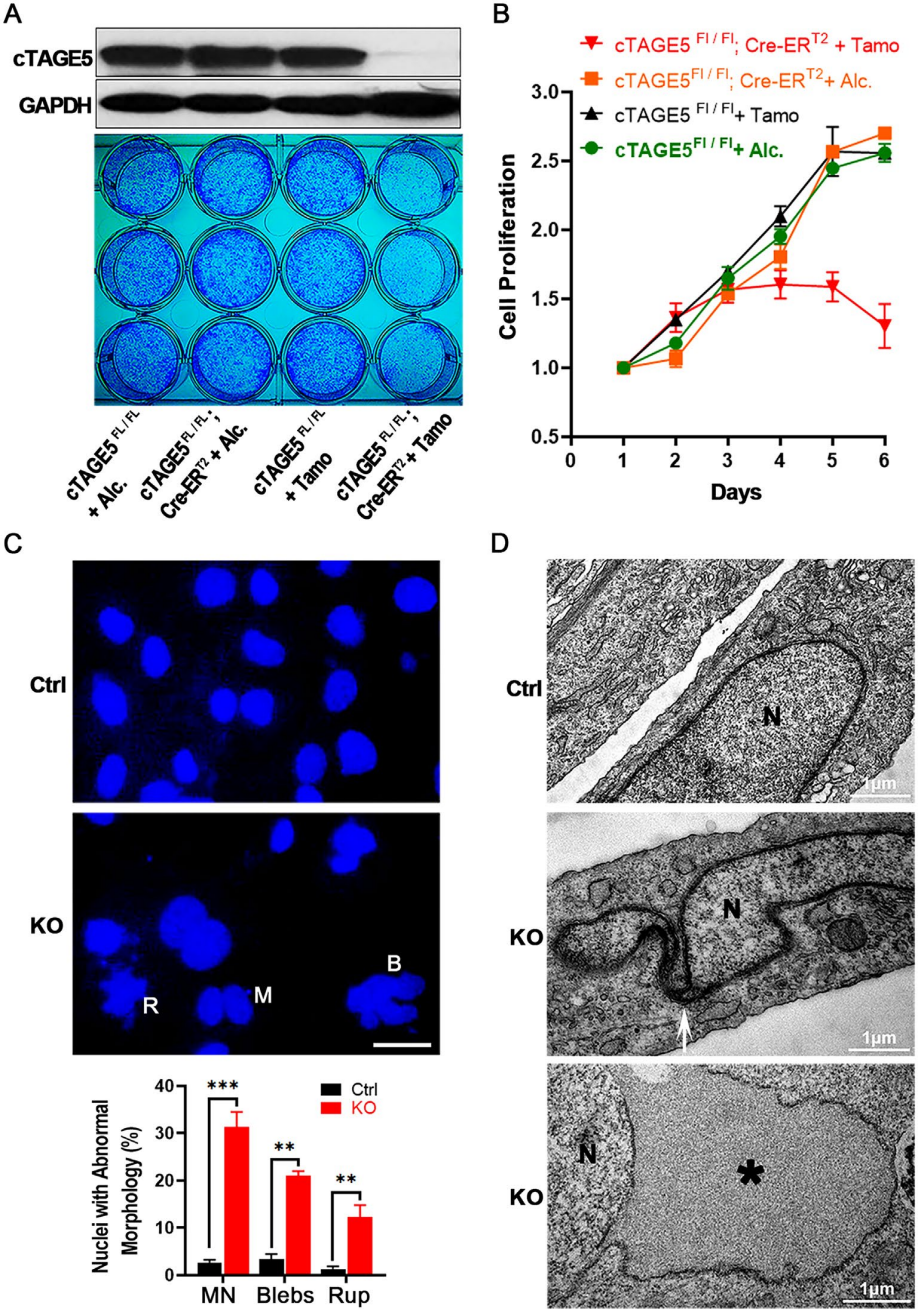
*cTAGE5*‐KO results in defects in cell proliferation and nuclear morphology in MEF cells. (A) 10^5^ MEF cells were seeded in a 12‐well plate. Ethanol or tamoxifen was applied for 3 days, followed by a 4‐day growth period. Depletion of *cTAGE5* was confirmed by western blot (top). Cells were also stained with crystal violet (bottom). (B) Cell growth was assessed using the MTT assay with O.D. at 570 nm (*n* = 3 independent experiments; mean ± SEM). (C) Nuclear morphology was examined via DAPI staining. B, blebs; M, micronuclei; R, ruptures. Statistical analysis was performed using the two‐tailed unpaired Student's *t*‐test, *n* = 1000 Ctrl cells and 1000 KO cells; mean ± SEM, *p < 0.05, **p < 0.01, ***p < 0.001. Scale bar, 10 μm. (D) Transmission electron microscopy revealed nuclear envelope defects (arrow) and outer nuclear membrane expansion (star) in *cTAGE5*‐KO MEF cells. N, nucleus.

To investigate the mechanism by which cTAGE5 influences cell fate, we generated conditional knockout mice of *cTAGE5* ^*FL/FL*^; *Cre‐ER* ^*T2*^ by crossing *cTAGE5* ^*FL/FL*^ mice with *Cre‐ER* ^T2^ transgenic mice. Western blot analysis confirmed the efficient knockout of cTAGE5 in *cTAGE5* ^*FL/FL*^; *Cre‐ER* ^*T2*^ MEF cells treated with tamoxifen (referred to as KO), but not in *cTAGE5* ^*FL/FL*^ and *cTAGE5* ^*FL/FL*^; *Cre‐ER* ^*T2*^ cells treated with ethanol, or *cTAGE5* ^*FL/FL*^ cells treated with tamoxifen (referred to as Ctrl) (Figure 1A, top). Cells were cultured in 12‐well plates for 3 days, treated with ethanol (vehicle) or 1 μM tamoxifen for 4 days, and then subjected to crystal violet staining. The results showed a significant reduction in the growth density of KO MEF cells (Figure 1A, bottom). Consistent with this, MTT assays demonstrated that cell proliferation was inhibited in KO MEF cells after 3 days of tamoxifen treatment (Figure 1B). DAPI staining revealed markedly abnormal nuclear morphology, accompanied by the presence of micronuclei, blebs, and ruptures in KO MEF cells (Figure 1C). Statistical analysis indicated that the proportion of cells with abnormal nuclei was as high as 65% (micronuclei 31.3%, blebs 21%, and ruptures 12.3%). Transmission electron microscopy showed prominent nuclear abnormalities and outer nuclear membrane expansion in KO MEF cells (Figure 1D).

### 2.2 Activation of the P53/P21 Senescence Pathway in *cTAGE5* Knockout MEF Cells

**FIGURE 2:**
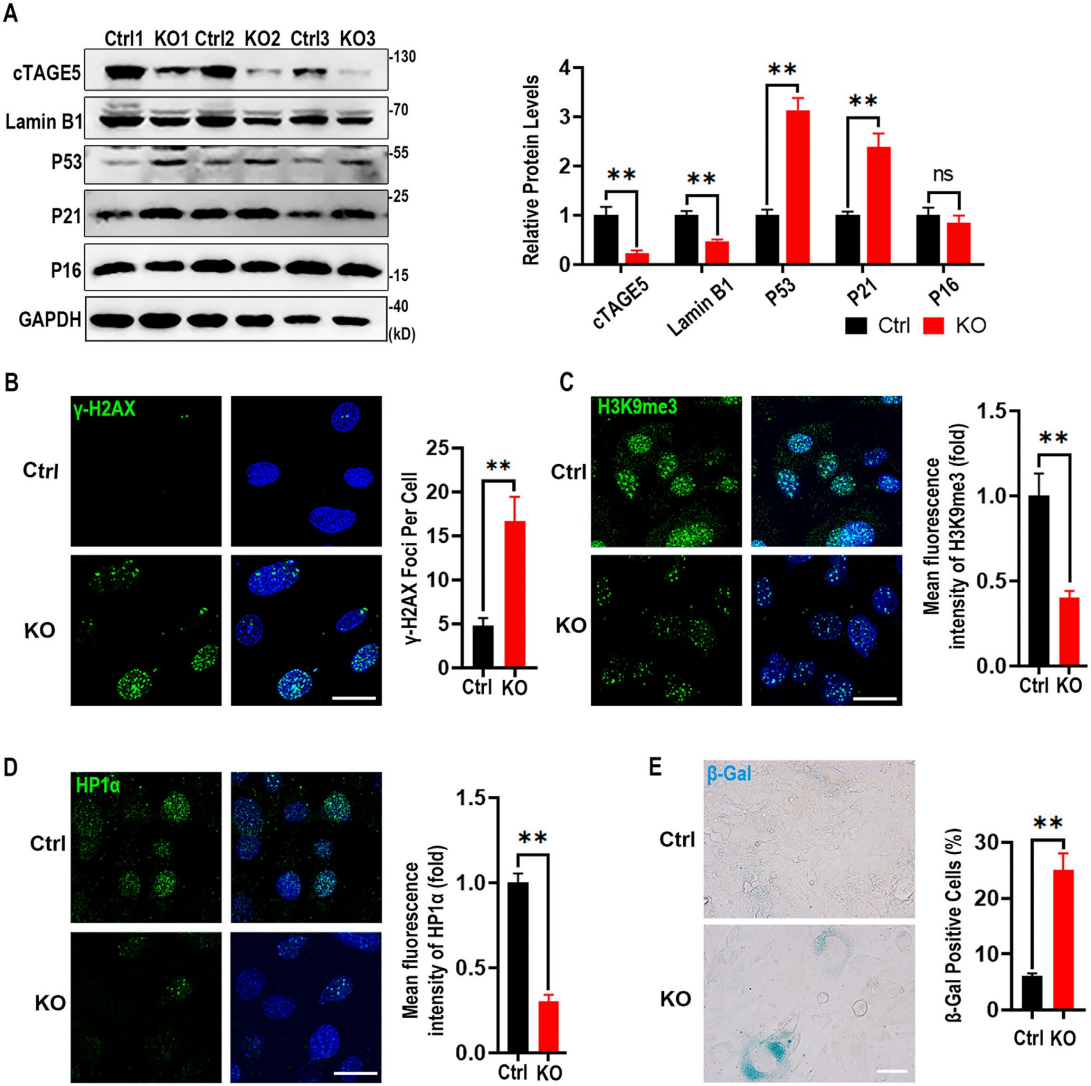
Loss of *cTAGE5* leads to cell senescence in MEF cells. (A) Western blot analysis of lysates from Ctrl or *cTAGE5*‐KO MEF cells (*n* = 3 independent experiments). (B) γ‐H2AX staining and foci quantification (*n* = 100 cells). The nuclei were stained with DAPI. (C) H3K9me3 staining shown on the left; H3K9me3‐positive cells are quantified as fold changes of their fluorescent intensity (*n* = 100 cells). (D) Immunofluorescence staining of HP1α and positive cells are quantified as fold changes of their fluorescent intensity (*n* = 100 cells). (E) MEF cells were cultivated in a 12‐well plate to an appropriate density and stained for β‐galactosidase (β‐Gal) activity. (*n* = 6 wells; mean ± SEM). Statistical analysis in (A)–(E): Two‐tailed unpaired Student's *t*‐test. ns, not significant; **p < 0.01. Scale bars, 10 μm.

Since that *cTAGE5* knockout leads to defects in nuclear morphology and cell proliferation, we proceeded to investigate whether *cTAGE5* deficiency results in cell senescence. After adding tamoxifen to the cell culture medium for 3 days, cTAGE5 protein levels in *cTAGE5* ^*FL/FL*^; *Cre‐ER* ^*T2*^ MEF cells (KO MEF) were significantly reduced compared to control *cTAGE5* ^*FL/FL*^ MEF cells (Ctrl MEF). This was accompanied by a marked decrease in Lamin B1, a biomarker associated with senescence, and a significant increase in the expression of the P53 and P21 proteins, but not P16 (Figure 2A). Immunostaining revealed that the *cTAGE5* KO MEF cells exhibited significantly enhanced γ‐H2AX staining signals and attenuated signals for heterochromatin hallmarks H3K9me3 and HP1ɑ compared to the control cells (Figure 2B–D). In line with these observations, we also noticed the increased accumulation of P21 staining signals and the senescence‐associated secretory phenotype (SASP)‐associated factors (Yang et al. 2024), including interleukin‐1β (IL1β) and interleukin‐6 (IL6) (Figure S1A–C). Live cell β‐galactosidase staining revealed a substantial increase in the number of positive cells (Figure 2E). These results indicate that the defective cell proliferation observed in *cTAGE5* KO MEF cells is due to the activation of the P53/P21 senescence pathway. In order to assess whether cTAGE5 loss has broad or tissue‐specific effects on cell senescence, we went on to evaluate the senescence hallmarks in primary cultured hepatocytes. We also found an accompanying increase in genomic and epigenomic instability in hepatocytes, such as increased DNA damage (marked by γH2AX increase), loss of heterochromatin (marked by H3K9me3 reduction), cell cycle arrest (marked by P21 increase), and activation of SASP (marked by IL1β, IL6, and TNFɑ) (Yang et al. 2024), as well as increased β‐gal positive cells (Figure S2). The results supported that senescence also occurred in the KO hepatocytes, indicating that cTAGE5 loss has broad effects on senescence. Our previous study demonstrated that cTAGE5 KO hepatocytes led to ER stress due to VLDL transport deficiency (Wang et al. 2016). To assess whether MEF senescence is caused by ER stress, we further tested ER stress in MEF cells. The results showed that ER stress markers Bip and Chop were not upregulated, and the spliced form of XBP1 mRNA was comparable between control and KO MEF cells (Yoshida et al. 2001) (Figure S3). We speculated that ER stress did not have a causative effect on the senescence phenotype, at least for MEF cells.

### 2.3 Identification of Lamin B Receptor (LBR) as a cTAGE5 Interacting Protein

**FIGURE 3:**
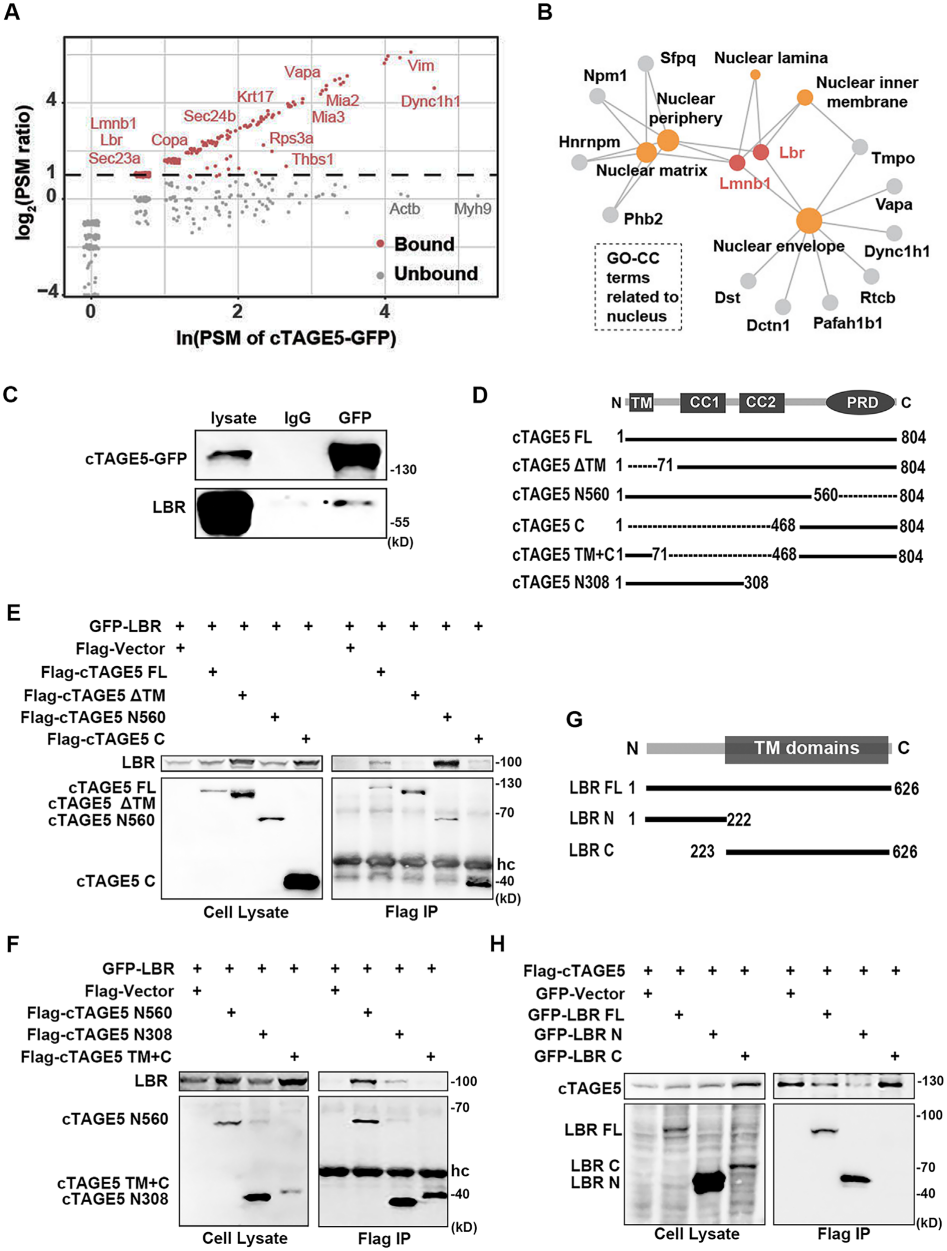
cTAGE5 interacts with the N‐terminal region of LBR through its N‐terminal domains. (A) Potential cTAGE5‐interacting proteins identified through IP‐MS analysis. PSM, peptide‐spectrum matches. (B) The significantly enriched Cellular Component terms related to nucleus (adjusted *p* < 0.05, BH method). (C) Co‐IP of *cTAGE5‐EGFP‐KI* MEF cell lysates using the anti‐GFP antibody, followed by immunoblotting of precipitates using anti‐GFP and anti‐LBR antibodies. (D) Schematic representation of full‐length (FL) cTAGE5 and its truncated mutants. CC, coiled‐coil domain; PRD, proline‐rich domain; TM, transmembrane domain. (E) and (F) HEK293 cells were co‐transfected with GFP‐LBR and either Flag‐tagged FL or cTAGE5 mutants, as indicated. After 24 h, cell lysates were subjected to precipitation with Flag agarose beads, and the immune complexes were detected using anti‐Flag or anti‐GFP antibodies. (G) Schematic of FL LBR and its N‐terminal and C‐terminal truncations. TM, transmembrane domain. (H) The interaction of cTAGE5 with FL LBR or its truncations was assessed by co‐IP. HEK293 cells were co‐transfected with Flag‐cTAGE5 and either GFP‐tagged FL or truncated LBR, as indicated and assessed as in E, F.

To elucidate the molecular mechanism by which *cTAGE5* KO leads to nuclear abnormalities and cell senescence, we proceeded to construct a *cTAGE5‐EGFP‐KI* mouse line using CRISPR/Cas9 and isolated MEF cell lines. Western blot analysis confirmed the expression of cTAGE5‐EGFP fusion protein in *cTAGE5‐EGFP‐KI* MEF cells (Figure S4A,B). We performed co‐immunoprecipitation (co‐IP) using GFP antibody on *cTAGE5‐EGFP‐KI* MEF cell lysates to pull down proteins that might interact with cTAGE5. These proteins identified by mass spectrometry include several known cTAGE5 interacting proteins, such as MIA3 and SEC23A (Figure 3A). According to the GO enrichment analysis, several potential interacting proteins were found to be involved in the maintenance of the nuclear membrane structure, such as lamin B1 (LMNB1) and LBR (Figure 3B). Among them, the inner nuclear membrane protein LBR was chosen as a potential candidate for further study due to its mutation leading to nuclear membrane abnormalities and cell senescence (Nikolakaki et al. 2017).

We first confirmed the interaction between endogenous cTAGE5 and LBR through co‐IP experiments (Figure 3C). To further delineate the specific region of cTAGE5 that interacts with LBR, we designed FLAG‐tagged cTAGE5 constructs, including the full‐length (FL, 1–804) and various truncated mutants: ΔTM (aa71‐804), N560 (aa1‐560), C (aa468‐804), TM + C (the transmembrane domain fused with the C‐terminus), and N308 (aa1‐308) (Figure 3D). Each of these constructs was co‐transfected with GFP‐LBR into HEK293 cells, followed by co‐IP using the FLAG antibody. The results showed that cTAGE5‐FL and cTAGE5‐N560 interacted with LBR, while N308 exhibited weak interaction; however, cTAGE5‐ΔTM, cTAGE5‐C, and TM + C did not interact with LBR (Figure 3E,F). These results suggest that LBR interacts with the coiled‐coil domains of cTAGE5. Localization of cTAGE5 in the ER requires its transmembrane domain, which is also required for the interaction of cTAGE5 with LBR.

To pinpoint which region of LBR interacts with cTAGE5, we designed GFP‐tagged LBR constructs, including full‐length LBR (FL, aa1‐626), LBR N (aa1‐222), and LBR C (aa223‐626) (Figure 3G). These constructs were co‐transfected with FLAG‐cTAGE5 into HEK293 cells, respectively. Co‐IP analysis revealed that LBR FL and LBR N222 interacted with cTAGE5, while LBR C did not (Figure 3H).

Taken together, these results indicate that cTAGE5 primarily interacts with the N‐terminal region of LBR through its coiled‐coil domains.

### 2.4 cTAGE5 Regulates the Localization and Stability of LBR

**FIGURE 4:**
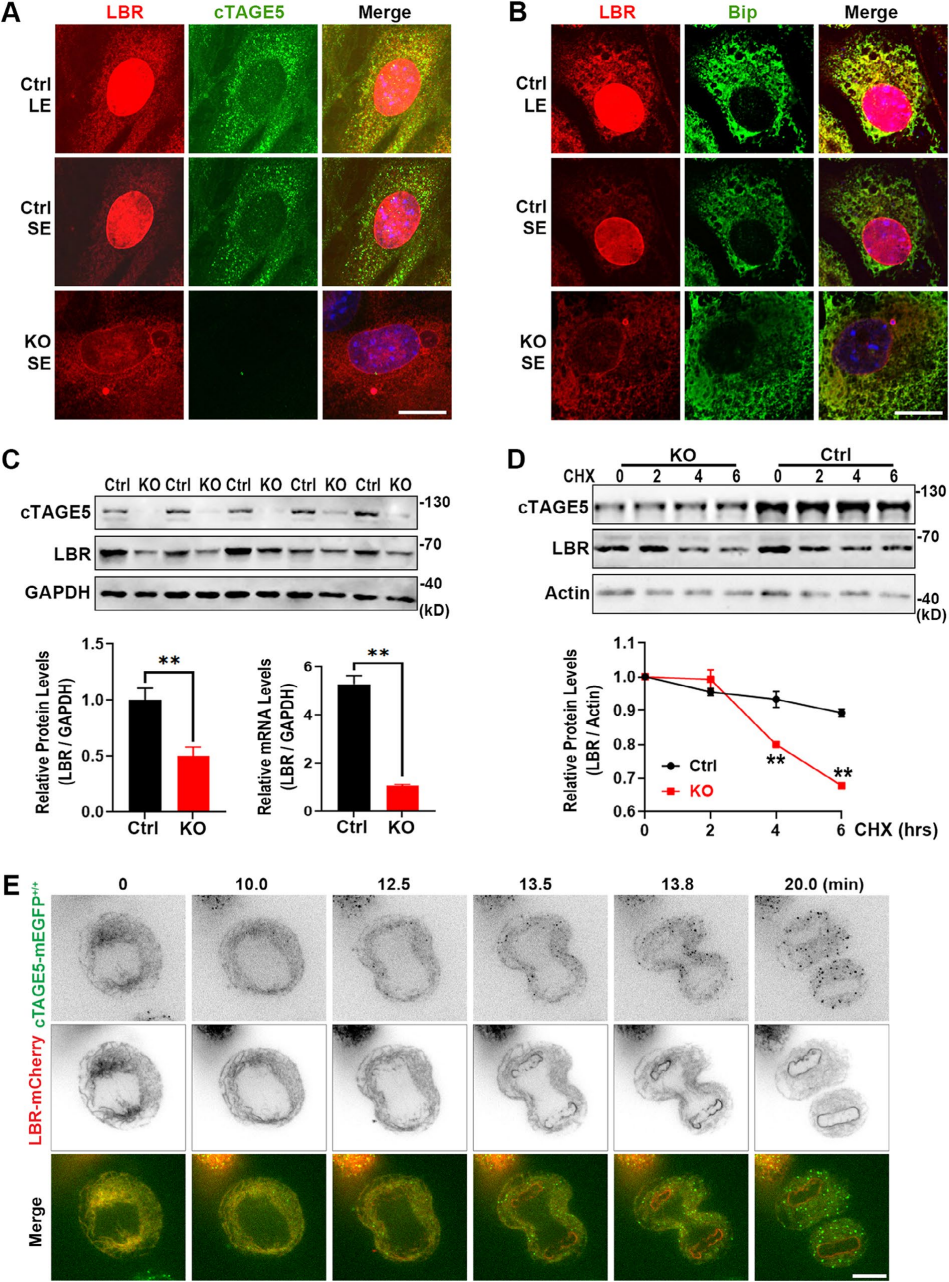
cTAGE5 regulates the localization and stability of LBR. (A) MEF cells were immunostained for LBR and cTAGE5. LE, long exposure; SE, short exposure. Note the significant increase in the cytoplasmic distribution of LBR in *cTAGE5* KO cells. (B) MEF cells were immunostained for LBR and Bip. Note the increased co‐localization of LBR with Bip in *cTAGE5* KO cells. (C) Western blot analysis of LBR expression in Ctrl and *cTAGE5* KO MEF cells (top panel). Quantification shows that LBR expression was significantly reduced in KO cells (bottom left panel; *n* = 3 independent experiments; mean ± SEM). RT‐qPCR analysis for mRNA levels of LBR was shown on the bottom right panel. (D) Western blot analysis of LBR expression in Ctrl and *cTAGE5* MEF KO cells treated with the protein synthesis inhibitor CHX (top panel). Quantification shows that LBR degradation was significantly faster in KO cells compared to Ctrl cells (bottom panel; *n* = 3 independent experiments; mean ± SEM). Statistical analysis in (C) and (D) was performed using the two‐tailed unpaired Student's *t*‐test. **p < 0.01. (E) Gene‐edited *cTAGE5‐mEGFP* ^+/+^ SUM159 cells were transfected with LBR‐mCherry and then imaged by spinning‐disk confocal microscopy. The montage shows the distribution of LBR and cTAGE5 during the cell cycle. Scale bars, 10 μm. [Correction added on 7 August 2025 after first online publication: Figure 4 has been replaced in this version.]

Since cTAGE5 and LBR interact with each other, we went on to elucidate the effect of *cTAGE5* deficiency on LBR by immunostaining MEF cells with cTAGE5 and LBR antibodies. Consistent with previous reports, LBR is primarily localized to the nuclear envelope (Figure 4A, middle). However, in *cTAGE5* KO MEF cells, the localization of LBR on the nuclear envelope was significantly reduced, with the majority found outside the nucleus (Figure 4A, bottom). Intriguingly, with prolonged exposure, a small amount of LBR was also observed to co‐localize with cTAGE5 in WT cells (Figure 4A, top). The LBR distributed in the cytoplasm mainly co‐localized with the ER marker Bip (Figure 4B). In KO MEF cells, not only was the localization of LBR altered, but its protein expression levels, as well as mRNA levels, were also significantly reduced (Figure 4C). When protein synthesis was inhibited with cycloheximide (CHX), LBR degradation was significantly accelerated (Figure 4D), indicating that cTAGE5 is involved in both LBR localization and protein stability.

### 2.5 cTAGE5 Co‐Localizes With LBR Throughout the Cell Cycle

Given that cTAGE5 colocalizes and interacts with LBR, we proceeded to track the localization of cTAGE5 during cell proliferation by generating the *cTAGE5‐mEGFP* knock‐in cell lines using CRISPR/Cas9. This enabled us to monitor the distribution of endogenous cTAGE5 through immunostaining or live cell imaging. Western blot analysis confirmed the homozygous replacement of cTAGE5 with cTAGE5‐mEGFP in the clonal breast cancer SUM159 cells (Figure S4C). Imaging cTAGE5‐mEGFP^+/+^ SUM159 cells using spinning‐disk confocal microscopy revealed the expected punctate distribution of cTAGE5‐mEGFP, which co‐localized with expressed TagRFP‐Sec23A or mScarlet‐I‐Sec16A, in line with previous reports (Wang et al. 2016; Li et al. 2022) (Figure S5A). Surprisingly, we also observed a weak cTAGE5‐mEGFP signal on the structure of the ER, which co‐localized with the ER marker mScarlet‐I‐Sec61β (Figure S5B). Additionally, as the cells entered mitosis and ERES disassembled, the cTAGE5‐mEGFP puncta disappeared and showed a predominantly distribution in the ER (Figure S5B, bottom). By overexpressing fluorescently tagged Rtn4a (a marker for ER tubules) or Climp63 (a marker for ER sheets) (Perkins and Allan 2021) in cTAGE5‐mEGFP^+/+^ SUM159 cells, we observed co‐localization of endogenous cTAGE5 with both Rtn4a and Climp63 (Figure S5C,D). The localization of cTAGE5 at both ERES and ER structures (both tubules and sheets) suggests that cTAGE5 may have additional functions beyond its established role in COPII transport.

Next, we tracked the dynamic distribution of cTAGE5 and LBR throughout the cell cycle in cTAGE5‐mEGFP^+/+^ SUM159 cells expressing LBR‐mCherry. During metaphase, the LBR‐mCherry signal co‐localized well with cTAGE5‐mEGFP on ER. However, during anaphase and telophase, while cTAGE5 began to reassemble at ERES, LBR started to accumulate at the nuclear envelope. Some residual LBR signal in the cytoplasmic ER continued to colocalize with cTAGE5 (Figure 4E).

**FIGURE 5:**
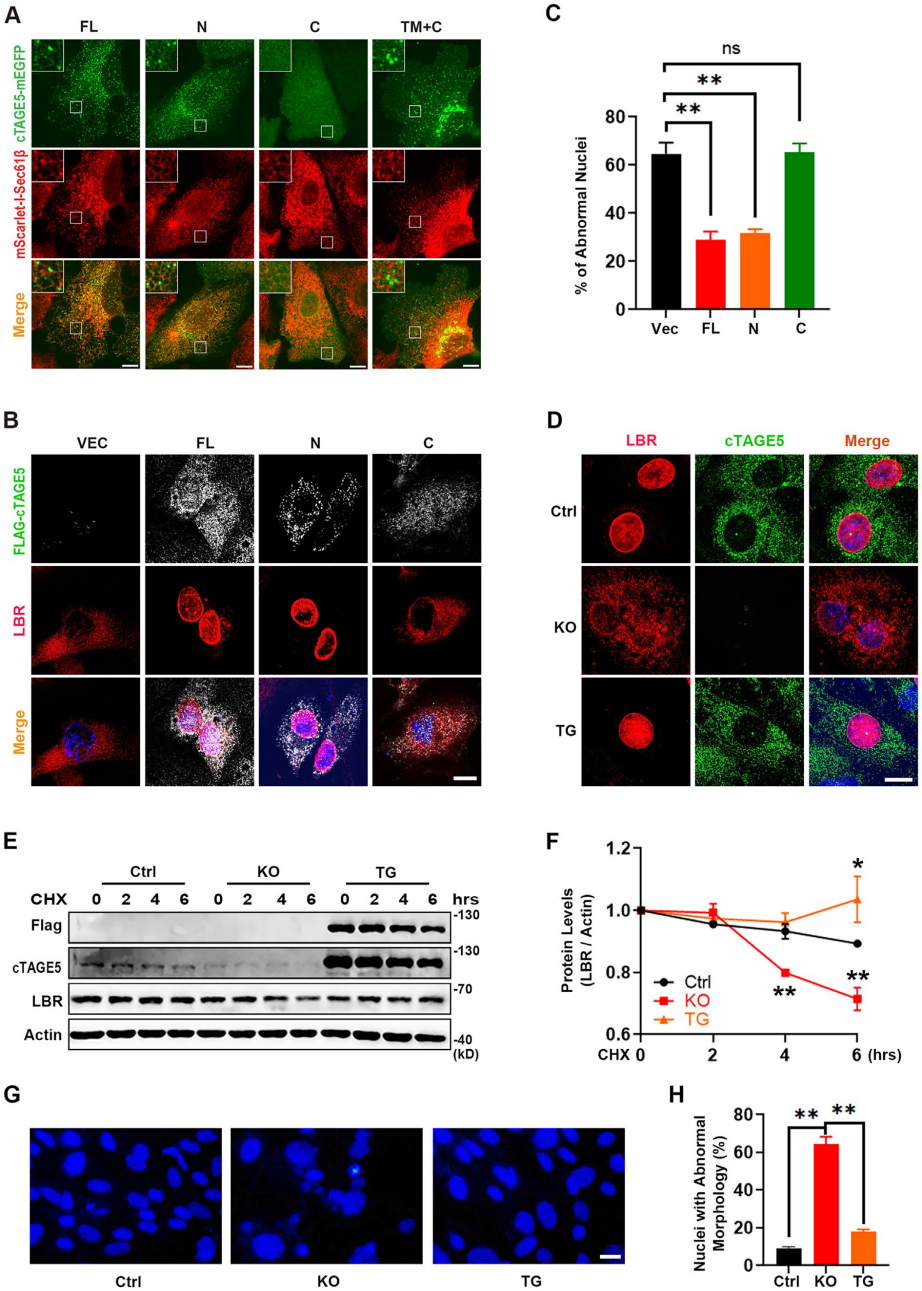
Restoration of normal LBR localization and nuclear morphology in *cTAGE5* KO MEF cells. (A) Gene‐edited *mScarlet‐I‐Sec61β* ^+/+^ SUM159 cells were transfected with either mEGFP‐tagged full‐length (FL) or cTAGE5 mutants and visualized using spinning‐disk confocal microscopy. (B) FLAG‐tagged FL or cTAGE5 mutants was transfected into *cTAGE5* KO MEF cells. The cTAGE5 and LBR proteins were immunostained using anti‐FLAG (gray) and LBR (red) antibodies, respectively, and the nucleus was stained with DAPI. The cells were then imaged using confocal microscopy. (C) The imaging results from Figure 6B are quantified. The proportions of cells with abnormal nuclei for the four groups are presented (*n* = 300 cells per group; mean ± SEM). **p < 0.01. (Two tailed unpaired Student's *t*‐test). (D) Ctrl, KO, and TG MEF cells were immunostained for LBR and cTAGE5. (E) Western blot analysis of Ctrl, KO, and TG MEF cells treated with the protein synthesis inhibitor CHX. (F) Quantification shows that the degradation of LBR in KO cells was rescued in TG cells (*n* = 3 independent experiments; mean ± SEM). *p < 0.05, **p < 0.01. (G) Nuclear morphology was examined using DAPI staining. (H) Statistical analysis of the ratio of nuclei with abnormal morphology (*n* = 1000 cells per group; mean ± SEM). *p < 0.05, **p < 0.01 (Two tailed unpaired Student's *t*‐test). Scale bars, 10 μm.

As cTAGE5 interacts with LBR primarily through its N560 domain (Figure 3), we next aimed to determine whether this interaction is crucial for maintaining LBR localization at the nuclear envelope. We generated genome‐edited SUM159 cells expressing mScarlet‐I‐Sec61β^+/+^ and then transfected the cells with either mEGFP‐tagged full‐length cTAGE5 or its mutants. We observed that both the full‐length cTAGE5 and its N560 fragment co‐localized with Sec61β, whereas the C‐terminus and TM + C (the transmembrane domain fused with the C‐terminus) mutants did not (Figure 5A). When these mutants were overexpressed in *cTAGE5* KO MEF cells, the full‐length cTAGE5 and its N560 fragment restored LBR localization on the nuclear envelope, as expected (Figure 5B). Furthermore, the proportion of cells with abnormal nuclei in *cTAGE5* KO MEF cells decreased from approximately 65% to about 30% (Figure 5C). These results indicate that LBR distribution at the nuclear envelope is regulated by the interaction between LBR and the N‐terminal 560aa (coiled‐coil domains) of cTAGE5.

More importantly, we generated a transgenic mouse line (TG) with human *cTAGE5* (*hcTAGE5*) cDNA inserted into ROSA26 loci (Zhang et al. 2018). Through immunostaining and Western blot analysis, we detected the expression of hcTAGE5 protein in MEFs (Figure 5D,E). Consistent with the results from the above in vitro experiments, *hcTAGE5* TG MEFs in the *cTAGE5* KO background exhibited normal nuclear localization and stability of LBR (Figure 5D–F). We also observed largely normal nuclear morphology in the *hcTAGE5* TG MEFs (Figure 5G,H). These results indicate that both the LBR localization and nuclear structure in KO MEF could be rescued by hcTAGE5.

These findings indicate that cTAGE5 interacts with LBR in ER and plays an important role in regulating LBR distribution and stability, in addition to its function in ER‐to‐Golgi transport.

### 2.6 Depletion of cTAGE5 Leads to Premature Aging in Adult Mice

**FIGURE 6:**
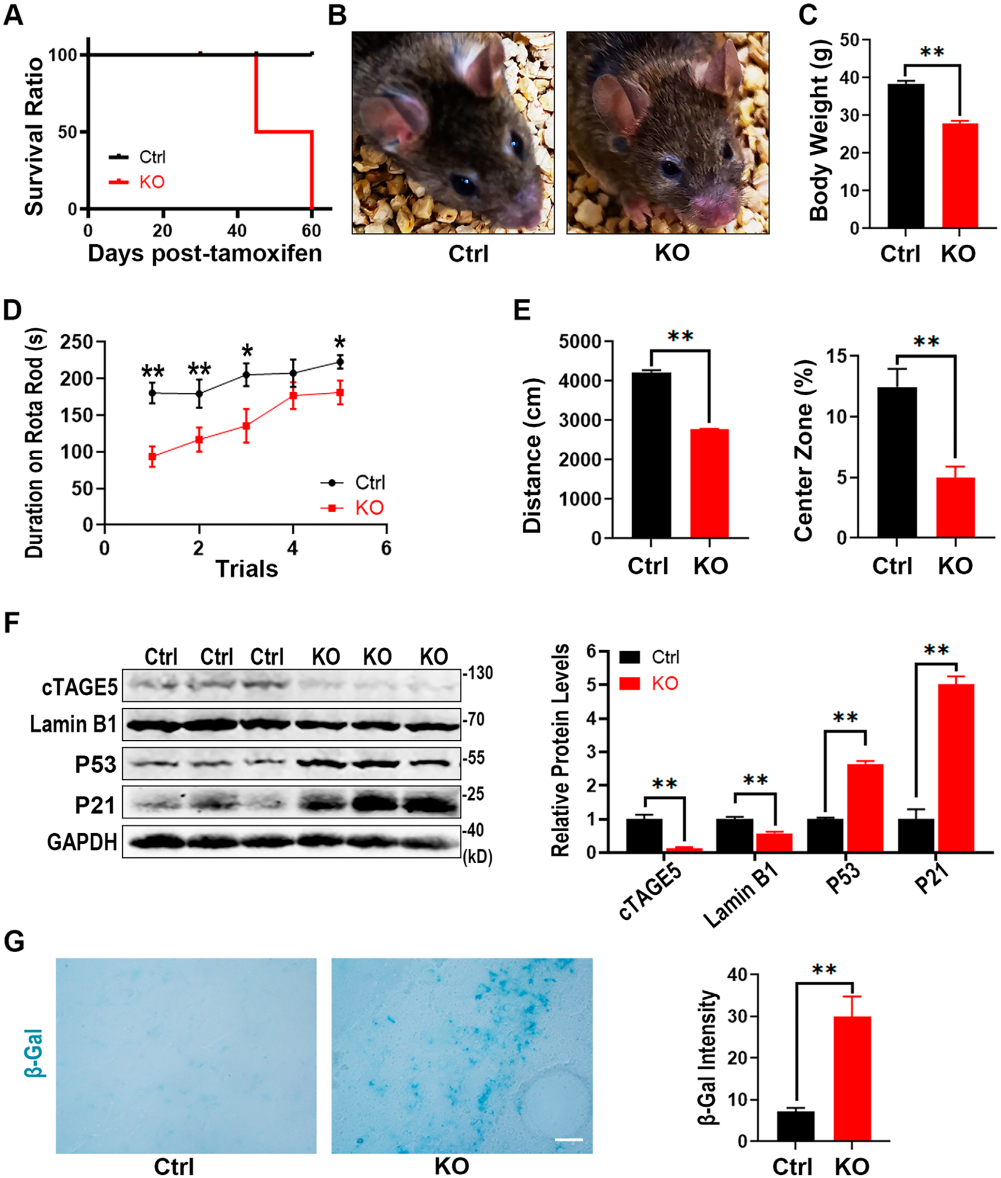
Loss of *cTAGE5* induces aging in mice. (A) Four‐month‐old mice were intraperitoneally injected with sesame oil or tamoxifen for two consecutive days. All tamoxifen‐injected mice died within 2 months (*n* = 8 mice per group). (B, C) Representative photographs of Ctrl and KO mice (B) and body weight measurements at 1 month after drug treatment (C) (*n* = 8 mice per group). (D) Rotarod tests showed a significant reduction in the time KO mice remained on the rotating rod (*n* = 8 mice per group). (E) Open field tests revealed a decrease in both total movement distance and time in the central zone by the KO mice (*n* = 8 mice per group). (F) Western blot analysis of cortical lysates, with quantification shown on the right (*n* = 3 independent experiments). (G) β‐Galactosidase staining of frozen brain cortex sections. The β‐Gal intensity was measured using Image J (3 sections per mouse, *n* = 4 mice per group; mean ± SEM). All data are presented as means ± SEM. Two‐tailed unpaired Student's *t*‐test. *p < 0.05; **p < 0.01. Scale bar, 10 μm.

Since conventional knockout of *cTAGE5* results in embryonic lethality, we further constructed a *cTAGE5* ^FL/FL^; Cre‐ER^T2^ mouse line to characterize the function of cTAGE5 in adult mice. Four‐month‐old mice were given intraperitoneal injections of either sesame oil (Ctrl) or tamoxifen (6 mg/40 g BW, KO) for two consecutive days and subsequently monitored daily. Survival curve monitoring revealed that all tamoxifen‐injected KO mice died within 2 months post‐treatment, whereas the control mice remained in normal physiological conditions (Figure 6A). One month after treatment, the KO mice showed significant signs of aging compared to control mice, including changes in hair color and density, eye brightness, and body weight (Figure 6B,C; Video S1). Further behavioral tests demonstrated that KO mice quickly fell off the rod in the rota‐rod test (Figure 6D) and showed significantly reduced movement distance and time in the center zone in the open field test (Figure 6E). These findings indicate that *cTAGE5* KO mice exhibit aging phenotypes similar to those previously reported in LBR KO mice (Lee et al. 2021).

One month after tamoxifen treatment, Western blot analysis of brain cortical lysates from KO and control mice showed significantly decreased cTAGE5 protein levels in the KO group, indicating the relatively effective knockout of *cTAGE5*. There was also a significant decrease in Lamin B1 levels, accompanied by a notable increase in P53 and P21 levels (Figure 6F). β‐galactosidase staining of cortical frozen sections revealed a significant increase in positive cells (Figure 6G). These results are consistent with those observed in *cTAGE5* KO MEF cells (Figure 2), indicating that senescence also occurs in the tissue of adult *cTAGE5* KO mice. To assess histological changes of different tissues, we performed H&E staining on tissue sections. No obvious physiological alterations were observed for brain, heart, or kidney. Notably, centralized nuclei were seen in muscle fibers. In addition, fibrotic areas in the liver tissue increased relative to that in the control liver, which is evidenced by sirius red staining (Figure S6). Moreover, we observed other senescence‐associated changes in muscle and liver, including attenuated signals for heterochromatin mark H3K9me3, as well as increased senescence‐associated secretory phenotype (SASP)‐associated factors (Figure S7). Taken together, although the overall appearance of brain, heart, and kidney was barely distinguishable from that of control, their physiological features might already change. The histological analysis revealed that muscle degeneration and liver fibrosis are more evident, as well as that the senescence hallmarks are altered. We speculated that multiple factors work together to lead to mice death after cTAGE5 KO.

As expected, significantly decreased levels of Lamin B1 and increased levels of P53 protein, as well as γ‐H2AX foci, were rescued in *hcTAGE5* TG MEF in the *cTAGE5* KO background, along with improved cell proliferation (Figure S8). These results indicate that, in addition to LBR localization and nuclear structure, cell senescence in KO MEF could be rescued by hcTAGE5.

## 3 Discussion

cTAGE5 has been demonstrated to play a crucial role in the secretion of various proteins by regulating ER‐to‐Golgi vesicle trafficking (Saito et al. 2011; Wang et al. 2016; Ma et al. 2022). In this study, we uncover that cTAGE5 is important for maintaining nuclear morphology, as well as for mouse development and body homeostasis. Mechanistically, we reveal that cTAGE5 regulates the trafficking of LBR between the ER and the nucleus.

Knocking out *cTAGE5* in zygotes results in severe developmental defects and lethality around E8.5, indicating that cTAGE5 is essential for development. This leads to our finding that *cTAGE5* KO MEF cells exhibit cellular senescence, likely due to abnormal nuclear morphology and formation of micronuclei (Figure 1). Those defects could be rescued by transgenic expression of human cTAGE5. The selective activation of P53/P21 without the upregulation of P16 suggests that MEF cells may preferentially activate the senescence response through P53/P21 rather than P16 in response to cTAGE5 loss. This illustrates that senescence is not a uniform process; instead, it can vary significantly depending on the type of stressor, cell type, and tissue context (Figure 2). Although cTAGE5's role in the assembly of the COPII complex has been reported, the absence of two crucial COPII components, Sec23A and Sec31, does not lead to cell senescence (Jin et al. 2012; Zhu et al. 2015), indicating that cTAGE5 has additional functions in cells. We previously demonstrated that Sec24D interacts with cTAGE5 (Wang et al. 2016), and deletion of *Sec24D* also leads to early embryonic lethality in mice (Baines et al. 2013). Whether this phenotype is related to senescence requires further investigation.

By generating the genome‐edited cells with endogenous cTAGE5 fluorescently tagged, we found that cTAGE5 localizes not only to the ERES but also to the ER tubules and sheets, particularly during metaphase when ERESs disassemble (Figure 4 and Figure S5). Through co‐IP analysis, we identified the interaction between cTAGE5 and LBR. Notably, in *cTAGE5* KO MEF cells, we observed a significant downregulation of LBR, which contributes to the membrane integrity of the nucleus (Figure 4). In addition to decreased protein levels, the localization of LBR is dramatically perturbed, with a substantial amount retained in the ER and a significant reduction in its presence at the nuclear membrane in *cTAGE5* KO cells (Figure 4).

We reveal that the distribution of LBR depends on its interaction with cTAGE5. While the full‐length and the N‐terminal 560 aa (including a transmembrane domain and two coiled‐coil domains) cTAGE5 are distributed on both the ER structure and ERES, cTAGE5 and LBR co‐localize exclusively on the ER structure. Thus, we speculate that cTAGE5 on the ER regulates LBR trafficking from the ER to the nucleus and maintains the localization of LBR at the inner nuclear membrane, as well as its protein stability. Consistent with this hypothesis, the full‐length and N‐terminal 560 aa of cTAGE5 (mainly two coiled‐coil domains) interact with LBR. Importantly, these interactions can effectively rescue nuclear membrane abnormalities in *cTAGE5* KO cells (Figure 5). The N‐terminal 222aa domain of LBR is exposed to the nucleoplasm and interacts with Lamin B and heterochromatin protein 1 (HP1) (Ma et al. 2007). Here, we found that this domain also mediates its interaction with cTAGE5 (Figure 3). During nuclear envelope assembly in telophase, LBR plays a pivotal role by recruiting nuclear envelope membrane vesicles to the chromatin surface, with the N‐terminal domain (aa 45–90) being crucial for its recruitment to the nuclear envelope membrane (Lu et al. 2010). Collectively, these results elucidate the critical role of cTAGE5 in maintaining nuclear envelope integrity, likely through its interaction with the N‐terminus of LBR.

It has been shown that the C‐terminal PRD domain of cTAGE5 binds to Sec23/24, and the coiled‐coil domain binds to TANGO1 for collagen VII secretion (Saito et al. 2011). We propose that the N‐terminal of cTAGE5 (especially the coiled‐coil domains) facilitates trafficking of LBR‐containing membranes from the ER to the nuclear envelope, which represents a unique mechanism for loading nuclear components into the nuclear envelope.

LBR knockdown has been shown to result in cellular senescence (Lu et al. 2010). Mutations in LBR can lead to Pelger‐Huët anomaly and Greenberg skeletal dysplasia. In cTAGE5 KO MEF cells and mouse cortical tissues, we detected significant downregulation of LBR, suggesting that the growth defects observed in MEF cells, along with embryonic lethality and premature aging in adult mice, may be due to senescence caused by mislocalization and downregulation of LBR. Consistent with this, downregulation of Lamin B1 and activation of the senescence pathway was detected in both cTAGE5 KO MEF cells and mouse tissues (Figures 2 and 6). Even though the global histological analysis did not reveal obvious alterations, many senescence hallmarks have been activated (Figures S6 and S7). The results supported that senescence also occurred in the KO muscle and liver tissues. All these above results supported that senescence occurred in multiple tissues due to cTAGE5 loss, indicating that cTAGE5 has a broad effect on aging.

LBR is an integral protein of the inner nuclear membrane, involved in chromatin organization and nuclear envelope stability. The nuclear lamina and associated proteins beneath the inner nuclear membrane also play a crucial role in maintaining nuclear structure, genomic stability, and gene expression regulation (Wong et al. 2022). Dysfunctions in these components are also involved in aging‐related diseases. For example, mutations in the LMNA gene, which encodes lamin A/C, can lead to a group of disorders known as laminopathies, including Hutchinson‐Gilford progeria syndrome (HGPS), characterized by accelerated aging (Solovei et al. 2013). Emerin is another nuclear envelope protein that interacts with lamin A/C. Mutations in emerin can cause Emery‐Dreifuss muscular dystrophy (Berk et al. 2013). Given the endoplasmic reticulum protein cTAGE5 is associated with aging by regulating the function of nuclear envelope‐associated protein LBR, we boldly speculate that cTAGE5 may play an important role in maintaining nuclear structure, genomic stability, and related signaling pathways. Especially as shown in the results of Figure 1C, nuclear abnormalities often manifest as micronuclei structures, which have been reported to be a significant cause of genomic instability leading to tumorigenesis (Adams et al. 2024). The cTAGE5 cDNA was initially cloned from the autoimmune serum antibodies of lymphoma patients (Heckel et al. 1997), suggesting the tumor‐relatedness of cTAGE5, which seems to confirm the anti‐aging function of the cTAGE5 protein. In the future, whether other endoplasmic reticulum structural proteins regulate cellular senescence through mechanisms similar to cTAGE5 remains a subject worthy of further investigation.

## 4 Conclusion

In addition to its critical role in ER‐to‐Golgi transport, cTAGE5 also regulates LBR trafficking between the ER and the nuclear membrane to maintain the integrity of both the ER and the nuclear envelope. *cTAGE5/MEA6* KO results in defects in LBR trafficking and cycling, leading to LBR mislocalization, reduced stability, and subsequent disruption of nuclear envelope integrity. This would compromise nuclear structure and genomic stability, leading to cell senescence. Therefore, cTAGE5 is essential for embryonic development and safeguards against aging.

## 5 Methods and Materials

### 5.1 Animals

*cTAGE5* ^*F/F*^ mice, with the insertion of two *loxp* sites flanking the 11th exon of the *cTAGE5* gene, were constructed as reported (Wang et al. 2016). FLAG‐*cTAGE5* transgenic mice were generated by Beijing Biocytogen Co. Ltd. (Zhang et al. 2018). All experimental procedures were performed according to protocols approved by the Institutional Animal Care and Use Committee at the Institute of Genetics and Developmental Biology, Chinese Academy of Sciences (No. AP2021004).

### 5.2 Primary MEF Cells and Hepatocytes Culture and Transfection

12.5‐day‐old mouse embryos with viscera and heads removed (E12.5) were dissected and digested with 0.25% (W/V) Trypsin–EDTA (GIBCO) for 5–10 min, and single‐cell suspensions were plated into 10 cm plates and cultured in DMEM medium (GIBCO) (10% FBS and 100 units of Penicillin/Streptomycin) at 37°C. For passaging MEFs, the cells are digested with Trypsin for 3 min. Plasmids were transfected into the primary MEFs using Lipofectamine 2000 (Invitrogen). Hepatocytes were isolated and cultured as before (Wang et al. 2016). Cells were treated with either ethanol (vehicle) or 1 μM tamoxifen for 3 consecutive days to induce *cTAGE5* KO.

### 5.3 Generation of the *cTAGE5‐mEGFP* ^+/+^ SUM159 Cells Using the CRISPR/Cas9 Approach

SUM159 cells were genome‐edited to incorporate mEGFP at the C‐terminus of cTAGE5 using the CRISPR/Cas9 approach as described (Ran et al. 2013). The single‐guide RNA (sgRNA) targeting human c*TAGE5/MEA6* (5′‐AAGAGAGCAAAAATATTGTC‐3′) was delivered as PCR amplicons. The donor construct used for homologous recombination was generated by cloning into the pUC19 vector with two ∼700‐nucleotide fragments of genomic DNA upstream and downstream of the stop codon of c*TAGE5* and the open reading frame of mEGFP. A (GGS)3 linker was inserted between the stop codon of c*TAGE5* and mEGFP. SUM159 cells were transfected with the donor plasmid and the free PCR product using Lipofectamine 3000 (Invitrogen) according to the manufacturer's instructions. Cells expressing mEGFP were enriched by fluorescence‐activated cell sorting (FACS) (FACSAria II, BD Biosciences). Cells expressing cTAGE5‐mEGFP were further subjected to single cell sorting to 96‐well plates. The gene‐edited monoclonal cells were confirmed by PCR, imaging, and Western blot analysis.

### 5.4 Immunostaining

Immunostaining was performed as described with minor modifications (Fan et al. 2017). Briefly, MEF cells or hepatocytes were fixed with cold methanol or PFA for 15 min at 4°C. The cells were incubated in blocking buffer (5% BSA, 0.1% Triton X‐100, 0.01% NaN_3_ in PBS) for 1 h at room temperature, and then incubated with the primary antibody (diluted in the blocking buffer) overnight at 4°C, and finally incubated with the secondary antibody conjugated with Alexa Fluor 488, 568, or 647 dyes (diluted in the blocking buffer) for 1 h at room temperature.

Tissue sections were deparaffinized, rehydrated, and subjected to antigen retrieval (microwave‐heated in sodium citrate buffer (pH 6.0) three times, each for 4 min). IF were performed with at least three mice for each genotype. IF sections were incubated with 0.3% Triton X‐100 for 10 min and then incubated with 5% BSA (albumin bovine serum) in PBS (phosphate buffered saline) for 1 h at room temperature, followed by incubation with a primary antibody for 1 h at room temperature or overnight at 4°C. After three washes with PBS, the sections were incubated with the secondary antibody conjugated with Alexa Fluor 488, 568, or 647 dyes (diluted in the blocking buffer) for 1 h at room temperature. The images were captured with a confocal laser scanning microscope (Zeiss LSM 800). For most of the immunofluorescence staining in this study, the results were quantified by calculating the percentage of positive cells using Image J. The result of immunofluorescence staining for H3K9me3 was performed with quantification of the immunofluorescence intensity for each cell using Image J.

The antibodies used for immunostaining were as follows: Bip (ab21685, Abcam, 1:2000), GFP (ab13970, Abcam, 1:1000), γ‐H2AX (05–636, millipore, 1:1000), cTAGE5/MEA6 (HPA000387, Sigma, 1:400), LBR (ab232731, Abcam, 1:2000), H3K9me3 (ab8898, Abcam, 1:500), HP1α (sc‐130446, Santa Cruz, 1:100), P21 (RLT3497, Ruiying, 1:100), IL6 (26404‐1‐AP, proteintech, 1:100), IL1β (sc‐52,012, Santa Cruz, 1:100), and TNFα (ab1793, Abcam, 1:100).

### 5.5 Co‐IP and Western Blotting

Co‐IP and Western blotting were performed as described with minor modifications (Wang et al. 2016). In brief, MEF cell lysates were prepared in the cell lysis buffer (20 mM Tris–HCl (pH 8.0), 50 mM NaCl, 10 mM HEPES (pH 7.4), 0.5% NP40, 0.5 mM EDTA (pH 8.0), Phosphatase Inhibitor Cocktail (Roche), 0.2 mM PMSF). For co‐IP of endogenous proteins, 2–3 μg of anti‐GFP antibodies (ab290, Abcam) were first incubated with Protein A/G agarose beads (15 μL + 15 μL) for 2 h at 4°C. The agarose beads were then incubated with 1 mg protein lysates of MEF cells at 4°C overnight. After washing with cell lysis buffer for at least 3 times, the proteins bound to the beads were eluted with SDS sample buffer and analyzed by western blotting. For Western blotting, the proteins in the cell lysates were separated by SDS‐PAGE and transferred to Nitrocellulose (NC) membranes. Then, the NC membranes were incubated with primary antibodies overnight at 4°C, followed by incubation with secondary antibodies conjugated with horseradish peroxidase. Finally, the enhanced chemiluminescence substrate was used to visualize the protein bands on the NC membrane. The antibodies used for western blotting were as follows: FLAG (M185, MBL, 1:2000), GAPDH (2118s, CST, 1:2000), cTAGE5/MEA6 (HPA000387, Sigma, 1:1000), Lamin B1 (ab16048, Abcam, 1:2000), P53 (10442‐1‐AP, Proteintech, 1:2000), P21 (ab109199, Abcam, 1:2000), P16 (ab211542, Abcam, 1:2000), GFP (ab13970, Abcam, 1:2000), LBR (ab232731, Abcam, 1:2000), and Actin (3700s, CST, 1:2000).

### 5.6 LC–MS/MS Analysis

For MS analyses, peptides were resuspended in 0.1% formic acid and analyzed using an LTQ Orbitrap Elite mass spectrometer (ThermoFisher Scientific) coupled online to an Easy‐nLC 1000 (Thermo Fisher Scientific) in data‐dependent mode. The peptides were separated by reverse phase liquid chromatography using a 150 μm (ID) × 250 mm (length) analytical column packed with C18 particles of 1.9 μm diameter. Precursor ions were measured in the Orbitrap analyzer at a resolution of 240,000 (at 400 m/z) and a target value of 10^6^ ions. The 20 most intense ions from each MS scan were isolated, fragmented, and measured in the linear ion trap. The CID normalized collision energy was set to 35.

### 5.7 Protein Identification

The data were analyzed using Thermo Scientific Proteome Discoverer software version 1.4. The proteome sequences for *Mus musculus* from UniProt were used for database searching, with the mass tolerance set to 0.05 Da. The false discovery rate was set to 0.01 for peptide and protein identifications. Cysteine carbamidomethylation and methionine oxidation were included in the search as the static modification and variable modification, respectively. The Sequest HT search engine calculates XCorr scores for peptide matches and provides the peptide matches having the best XCorr score for each spectrum. The Sequest HT node calculates the XCorr value for every peptide candidate. Spectral counts were used for the relative quantification of proteins between samples.

### 5.8 Gene Ontology Enrichment Analysis

The clusterProfiler package (v4.7.1) was used to perform Gene Ontology (GO) enrichment analysis in Rstudio. Potential cTAGE5‐interacting proteins with a log‐ratio of PSM (cTAGE5‐GFP vs. IgG) greater than 1 were selected as input. The significantly enriched Cellular Component terms (adjusted *p* < 0.05, BH method) were filtered to focus on those related to the nucleus. Proteins contributing to these terms were visualized using the cnetplot function.

### 5.9 Electron Microscopy and Histological Analysis

MEF cells were fixed in 2.5% glutaraldehyde in 0.1 M phosphate buffer (pH 7.4) overnight at 4°C, then scraped and centrifuged into a cell pellet. After three washes in phosphate buffer, the cell pellet was postfixed with 1% OsO_4_ in phosphate buffer for 2 h and washed three times in phosphate buffer. The cell pellet was then dehydrated in a graded series of acetone (30%, 50%, 70%, 80%, 90%, 95%, and 100%) for approximately 10–15 min at each concentration. The pellet was infiltrated with SPURR resin. Ultrathin sections were cut with a Leica ultramicrotome. After sequential staining with uranyl acetate and lead citrate, the sections were photographed using a transmission electron microscope (JEM‐1400, JEOL, Japan) operated at 80 kV, and images were captured with a CCD camera (Gatan).

For histological analysis, various tissues were dissected from mutant and control mice immediately after euthanasia and fixed in 4% paraformaldehyde for up to 24 h, then dehydrated in a graded series of ethanol and embedded in paraffin. 5 μm thick sections were cut with a Leica RM2255 Rotary Microtome. After deparaffinization, sections were stained with hematoxylin and eosin (H&E) and observed under a light microscope with a CCD camera. Sirius Red solution was used for the collagen staining to assess the degree of fibrosis.

### 5.10 β‐Galactosidase Staining

Cells cultured in a 12‐well plate or frozen brain sections were washed twice with 1× PBS to remove dead cells, followed by the addition of freshly prepared fixative (2% formaldehyde and 0.2% glutaraldehyde in PBS). The samples were then fixed at room temperature for 5 min. Following this, the samples were washed twice with 1× PBS to remove residual fixative. Next, 1 mL of freshly prepared β‐gal staining solution (citric acid/sodium phosphate buffer 40 mM, potassium ferricyanide 5 mM, potassium ferrocyanide 5 mM, NaCl 150 mM, MgCl_2_ 2 mM, and X‐gal 1 mg/mL in ddH_2_O) was added, and the samples were stained at 37°C in a bacterial incubator for 12 h. Following staining, the samples were washed twice with 1× PBS to remove residual staining solution and then imaged using an inverted phase contrast microscope.

### 5.11 Behavior Tests

The behavior tests were performed as previously described (Zhang et al. 2016). In brief, in the Rota‐Rod test, mice were put on the rotating rod of the Rota Rod treadmill set at 10 rpm, and the time spent by each mouse on the rotating rod was measured. For the open field test, mice were placed in a chamber (40 cm × 40 cm × 49 cm) with a white background, and their movements were observed and analyzed with SMART software. If a mouse's speed fell below 0.3 cm/s, it was considered to be at rest. The moving distance, speed, duration of movement, and duration of rest were recorded and measured during a 600‐s observation period.

### 5.12 Imaging and Data Analysis

Images of the mouse body and brain were taken using a Canon camera. Samples from β‐galactosidase staining and immunostaining were imaged using a Zeiss LSM700 confocal microscope. Live‐cell imaging of the cTAGE5‐mEGFP^+/+^ SUM159 cells was performed using a spinning‐disk confocal microscope. All images were analyzed with software of Photoshop or ImageJ/Fiji (NIH). For the quantification analysis, which was not performed blinded to the experimental conditions, a two‐tailed unpaired Student's *t*‐test was conducted using GraphPad Prism or Excel software. Significant differences were indicated as *p < 0.05, **p < 0.01, and ***p < 0.001.

## Author Contributions

Yaqing Wang and Pengyu Sun performed the experiments and analyzed the data; Fuqiang Yang and Tiantian Ma performed the MS analysis; Junwan Fan and Lei Shi improved the experimental methods; Nan Li performed immunofluorescence staining; Fei Gao provided the mice strain; Fei Gao, Kangmin He, and Zhiheng Xu conceived the study and designed the experiments. Yaqing Wang, Kangmin He, and Zhiheng Xu wrote the paper.

## Conflicts of Interest

The authors declare no conflicts of interest.

## Supporting information


**Video S1:** Loss of *cTAGE5* induces aging in mice. Four‐month‐old mice were given intraperitoneal injections of either sesame oil (Ctrl) or tamoxifen (6 mg/40 g BW, KO) for two consecutive days and subsequently monitored daily. This video revealed normal physiological conditions of Ctrl mice at 1 month after drug treatment. However, the KO mice exhibited significant signs of aging compared to control mice, including changes in hair color and density, eye brightness, and reduced movement.

**Figure S1:** Loss of cTAGE5 leads to cell senescence in MEF cells. (A) Immunofluorescence staining of P21 and the percentage of positive cells were quantified (*n* = 100 cells). (B, C) SASP markers were stained and quantified as above. Statistical analysis: two‐tailed unpaired Student's *t*‐test. ***p < 0.001; **p < 0.01. Scale bars, 10 μm.
**Figure S2:** Aging‐related loss of genomic and epigenomic stability in the primary cultured hepatocytes. (A) Immunofluorescent staining of cTAGE5 to indicate its efficient KO. (B) γ‐H2AX staining and foci quantification (*n* = 100 cells). (C) H3K9me3 staining shown on the left; H3K9me3‐positive cells are quantified as fold changes of their fluorescent intensity (*n* = 100 cells). (D) Immunofluorescence staining of P21 and the percentage of positive cells were quantified (*n* = 100 cells). (E–G) SASP markers were stained and quantified as above. (H) Hepatocytes were cultivated in a 12‐well plate to an appropriate density and stained for β‐galactosidase (β‐Gal) activity. (*n* = 6 wells; mean ± SEM). Statistical analysis: two‐tailed unpaired Student's *t*‐test. *p < 0.05; **p < 0.01. Scale bars, 10 μm.
**Figure S3:** ER stress is not activated in cTAGE5 KO MEF cells. (A) Splicing of XBP1 mRNA upon ER stress. MEF cells were treated with 1 μg/mL tunicamycin for the indicated time and total RNA prepared was amplified by RT‐PCR. Spliced versions of XBP1 cDNA was comparable between Ctrl and KO. Ns, not significant. (B) The protein levels of Bip and Chop, two ER stress markers, were not upregulated in KO MEF cells, indicating that ER stress was not induced. Statistical analysis: two‐tailed unpaired Student's *t*‐test. ns, not significant.
**Figure S4:** (A) Strategy for knocking in the EGFP/mEGFP tag into the *cTAGE5* genome using CRISPR/Cas9. (B) Characterization of *cTAGE5‐EGFP‐KI* MEF cells by Western blotting using antibodies against GFP (top) and cTAGE5 (bottom). (C) Characterization of *cTAGE5‐mEGFP* ^+/+^ SUM159 cells by Western blotting using antibodies against GFP (top) and cTAGE5 (bottom).
**Figure S5:** Subcellular distribution of cTAGE5. (A and B) Gene‐edited cTAGE5‐mEGFP+/+ SUM159 cells were transfected with the indicated plasmids and then imaged by spinning‐disk confocal microscopy. (C) cTAGE5‐mEGFP+/+ SUM159 cells were transfected with Rtn4a‐mScarlet3 or mScarlet3‐Climp63 and then imaged by spinning‐disk confocal microscopy. Plots showing the fluorescent intensity profile along the lines on the merged image. (D) cTAGE5‐mEGFP+/+ SUM159 cells were transfected with Rtn4a‐mScarlet3 or mScarlet3‐Climp63 and then imaged by spinning‐disk confocal microscopy. The montage shows the distribution of cTAGE5 with Rtn4a or Climp63 during the cell cycle. Scale bars, 10 µm.
**Figure S6:** H&E and Sirius red staining on tissue sections. (A) Assessment of histological alterations for brain, heart, or kidney. (B) H&E staining on muscle and liver tissue sections. Notably, centralized nuclei were seen in muscle fibers (arrow) and fibrotic areas in the liver tissue (arrow) increased relative to that in the control liver. (C) Sirius red staining revealed the fibrosis bridge on liver sections (arrow).
**Figure S7:** Loss of cTAGE5 leads to cell senescence in liver (A–D) and muscle (E, F) tissues. (A) Immunofluorescent staining of H3K9me3 shown on the left; H3K9me3‐positive cells are quantified as fold changes of their fluorescent intensity (*n* = 100 cells). (B–D) Immunofluorescence staining of SASP markers and the percentage of positive cells were quantified (*n* = 100 cells). (E) Immunofluorescent staining of H3K9me3 shown on the left; H3K9me3‐positive cells are quantified as fold changes of their fluorescent intensity (*n* = 100 cells). (F–H) Immunofluorescence staining of SASP markers and the percentage of positive cells were quantified (*n* = 100 cells). Statistical analysis: two‐tailed unpaired Student's *t*‐test. **p < 0.01. Scale bars, 10 μm.
**Figure S8:** Cell senescence and proliferation can be rescued by cTAGE5 overexpression in KO MEF cells. (A) Western blot analysis was performed on lysates from Ctrl, KO, and TG MEF cells (left). Protein expression levels were quantified (right; *n* = 3 independent experiments; mean ± SEM). ns, not significant; **p < 0.01 (Two tailed unpaired Student's *t*‐test). (B) and (C) γ‐H2AX staining and quantification. (*n* = 100 cells per group; mean ± SEM). Scale bar, 10 μm. (D) Cell growth was assessed using the MTT assay (*n* = 3 independent experiments; mean ± SEM).

## Data Availability

The raw data and result files of IP‐MS have been deposited to the ProteomeXchange Consortium (https://proteomecentral.proteomexchange.org) via the iProX partner repository (Chen et al. 2022) with the dataset identifier PXD063263. The other data needed to address the conclusions in this paper are present in the paper or the Supporting Information.
