# Supplementary material for: cTAGE5/MEA6 Regulates LBR Localization to Maintain Nuclear Envelope Integrity and Safeguard Against Aging
Source: Aging Cell. 2025 Jul 30;24(10):e70185. doi: 10.1111/acel.70185 (PMC12507394; doi:10.1111/acel.70185)
Supplement: Supplementary file 2 — Figure S1: Loss of cTAGE5 leads to cell senescence in MEF cells. (A) Immunofluorescence staining of P21 and the percentage of positive cells were quantified (n = 100 cells). (B, C) SASP markers were stained and quantified as above. Statistical analysis: two‐tailed unpaired Student's t‐test. ***p < 0.001; **p < 0.01. Scale bars, 10 μm. Figure S2: Aging‐related loss of genomic and epigenomic stability in the primary cultured hepatocytes. (A) Immunofluorescent staining of cTAGE5 to indicate its efficient KO. (B) γ‐H2AX staining and foci quantification (n = 100 cells). (C) H3K9me3 staining shown on the left; H3K9me3‐positive cells are quantified as fold changes of their fluorescent intensity (n = 100 cells). (D) Immunofluorescence staining of P21 and the percentage of positive cells were quantified (n = 100 cells). (E–G) SASP markers were stained and quantified as above. (H) Hepatocytes were cultivated in a 12‐well plate to an appropriate density and stained for β‐galactosidase (β‐Gal) activity. (n = 6 wells; mean ± SEM). Statistical analysis: two‐tailed unpaired Student's t‐test. *p < 0.05; **p < 0.01. Scale bars, 10 μm. Figure S3: ER stress is not activated in cTAGE5 KO MEF cells. (A) Splicing of XBP1 mRNA upon ER stress. MEF cells were treated with 1 μg/mL tunicamycin for the indicated time and total RNA prepared was amplified by RT‐PCR. Spliced versions of XBP1 cDNA was comparable between Ctrl and KO. Ns, not significant. (B) The protein levels of Bip and Chop, two ER stress markers, were not upregulated in KO MEF cells, indicating that ER stress was not induced. Statistical analysis: two‐tailed unpaired Student's t‐test. ns, not significant. Figure S4: (A) Strategy for knocking in the EGFP/mEGFP tag into the cTAGE5 genome using CRISPR/Cas9. (B) Characterization of cTAGE5‐EGFP‐KI MEF cells by Western blotting using antibodies against GFP (top) and cTAGE5 (bottom). (C) Characterization of cTAGE5‐mEGFP +/+ SUM159 cells by Western blotting using antibodies against GFP [file ACEL-24-e70185-s001.pptx]

## Slide 1
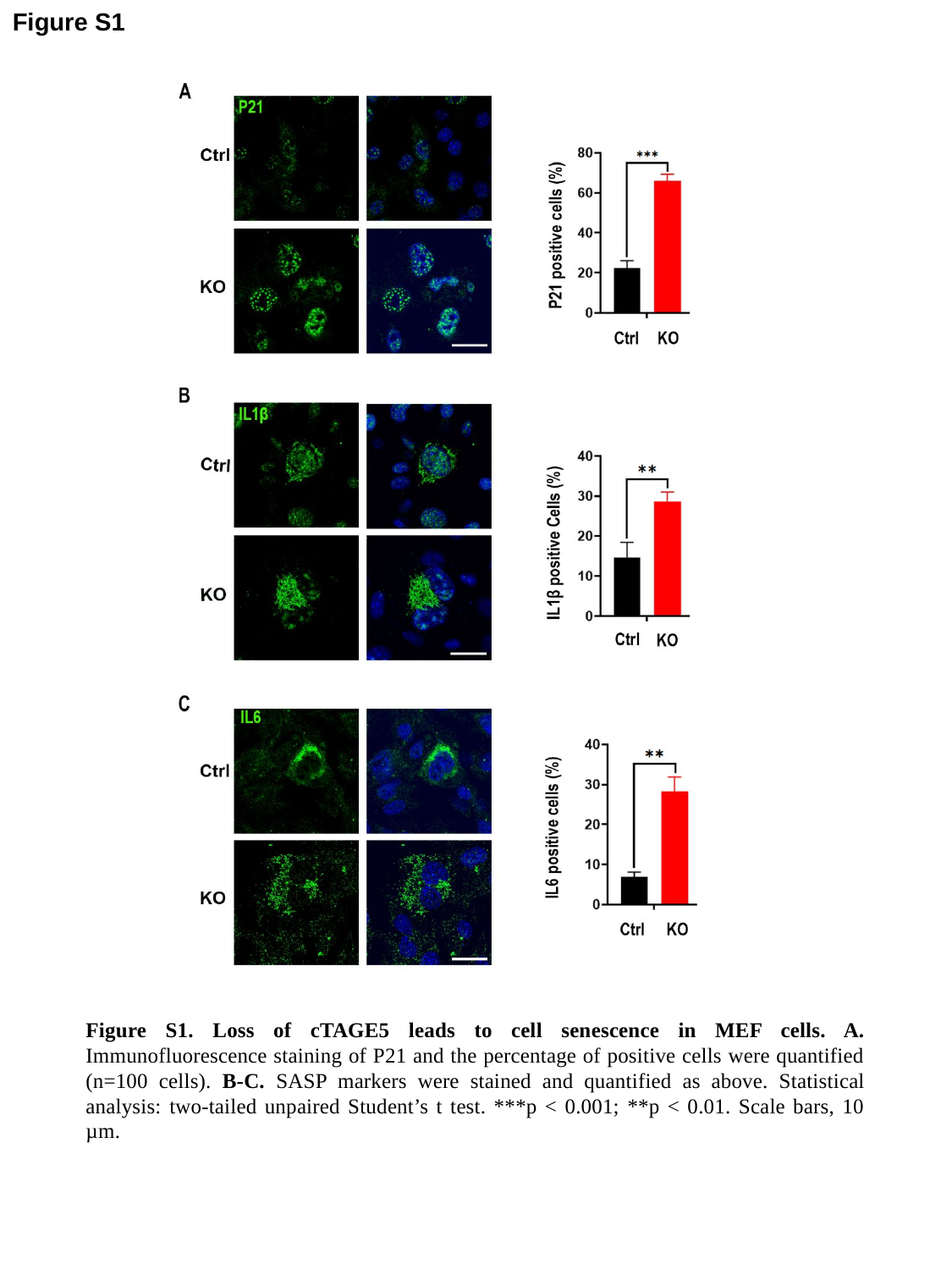

Figure S1
Figure S1. Loss of cTAGE5 leads to cell senescence in MEF cells. A. Immunofluorescence staining of P21 and the percentage of positive cells were quantified (n=100 cells). B-C. SASP markers were stained and quantified as above. Statistical analysis: two-tailed unpaired Student’s t test. ***p < 0.001; **p < 0.01. Scale bars, 10 µm.

## Slide 2
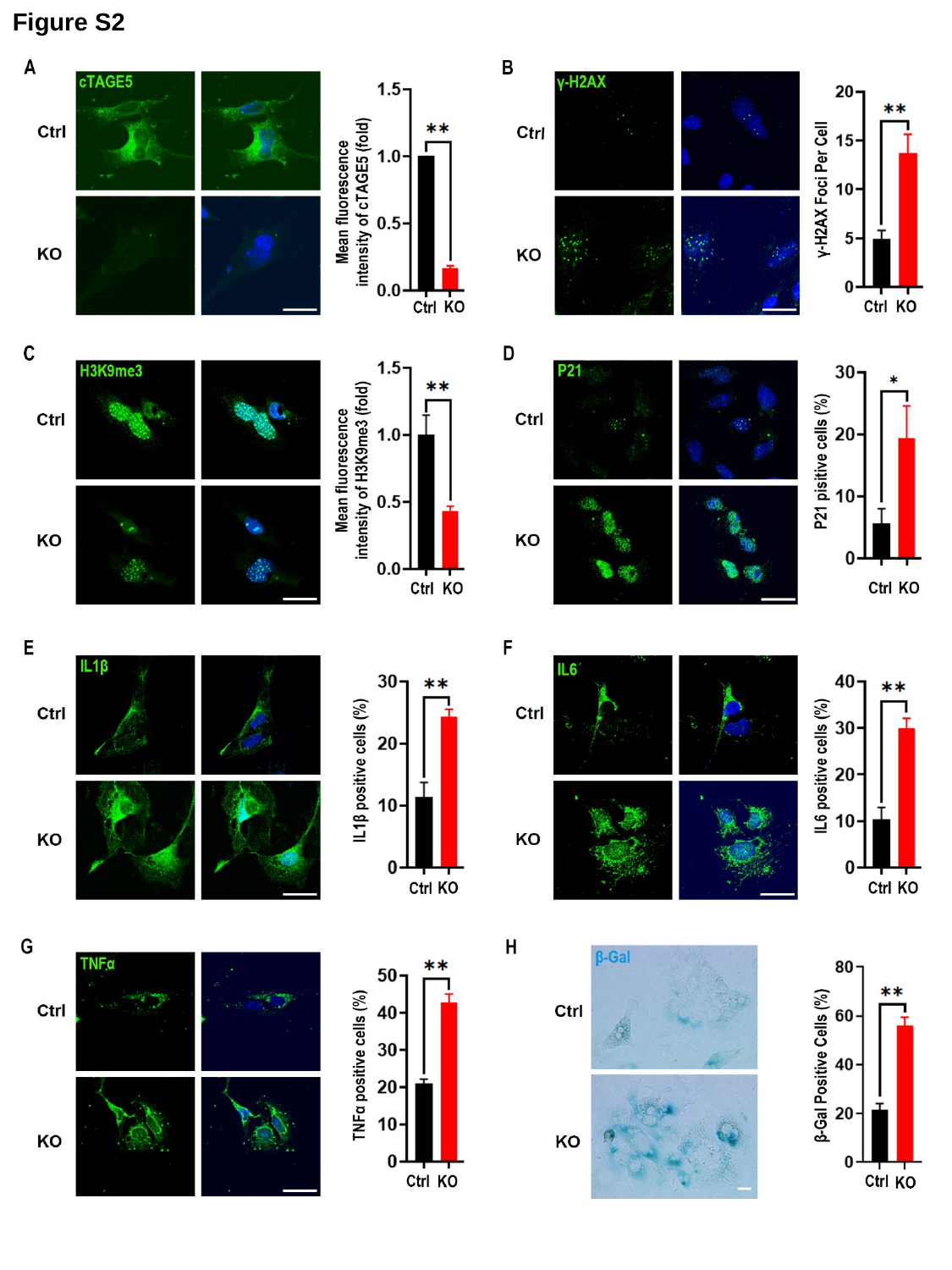

Figure S2

## Slide 3
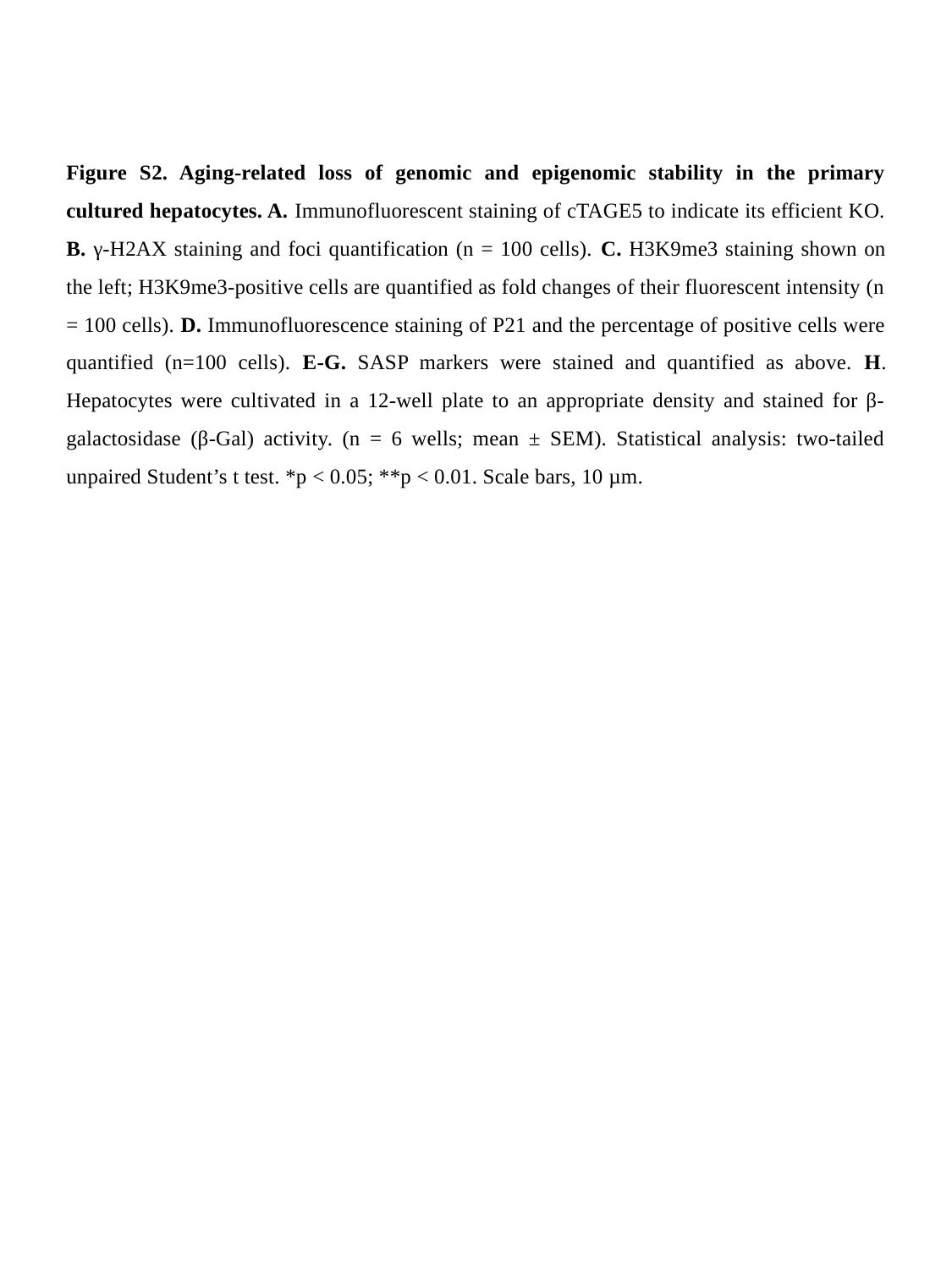

Figure S2. Aging-related loss of genomic and epigenomic stability in the primary cultured hepatocytes. A. Immunofluorescent staining of cTAGE5 to indicate its efficient KO. B. γ-H2AX staining and foci quantification (n = 100 cells). C. H3K9me3 staining shown on the left; H3K9me3-positive cells are quantified as fold changes of their fluorescent intensity (n = 100 cells). D. Immunofluorescence staining of P21 and the percentage of positive cells were quantified (n=100 cells). E-G. SASP markers were stained and quantified as above. H. Hepatocytes were cultivated in a 12-well plate to an appropriate density and stained for β-galactosidase (β-Gal) activity. (n = 6 wells; mean ± SEM). Statistical analysis: two-tailed unpaired Student’s t test. *p < 0.05; **p < 0.01. Scale bars, 10 µm.

## Slide 4
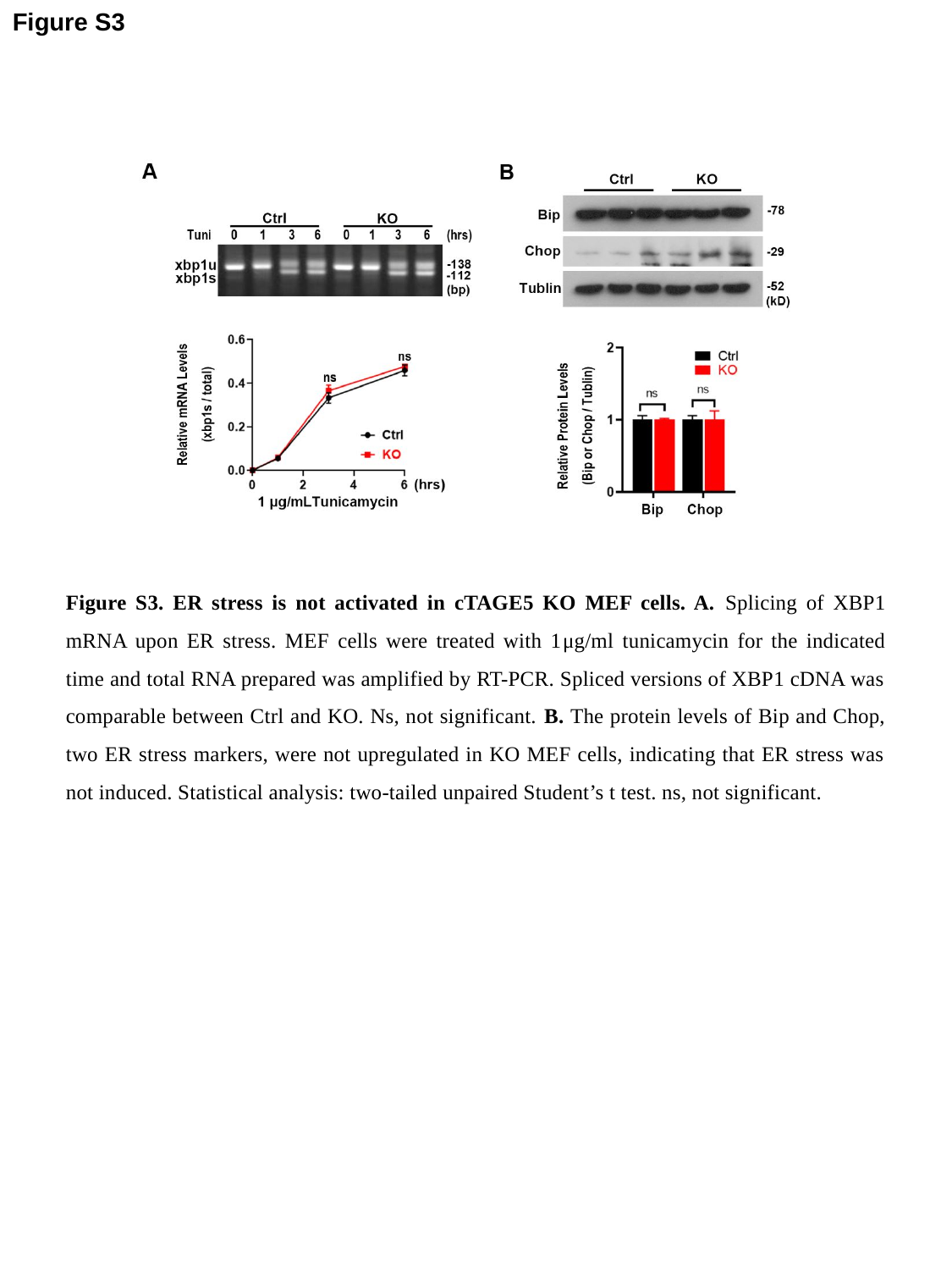

Figure S3
Figure S3. ER stress is not activated in cTAGE5 KO MEF cells. A. Splicing of XBP1 mRNA upon ER stress. MEF cells were treated with 1μg/ml tunicamycin for the indicated time and total RNA prepared was amplified by RT-PCR. Spliced versions of XBP1 cDNA was comparable between Ctrl and KO. Ns, not significant. B. The protein levels of Bip and Chop, two ER stress markers, were not upregulated in KO MEF cells, indicating that ER stress was not induced. Statistical analysis: two-tailed unpaired Student’s t test. ns, not significant.

## Slide 5
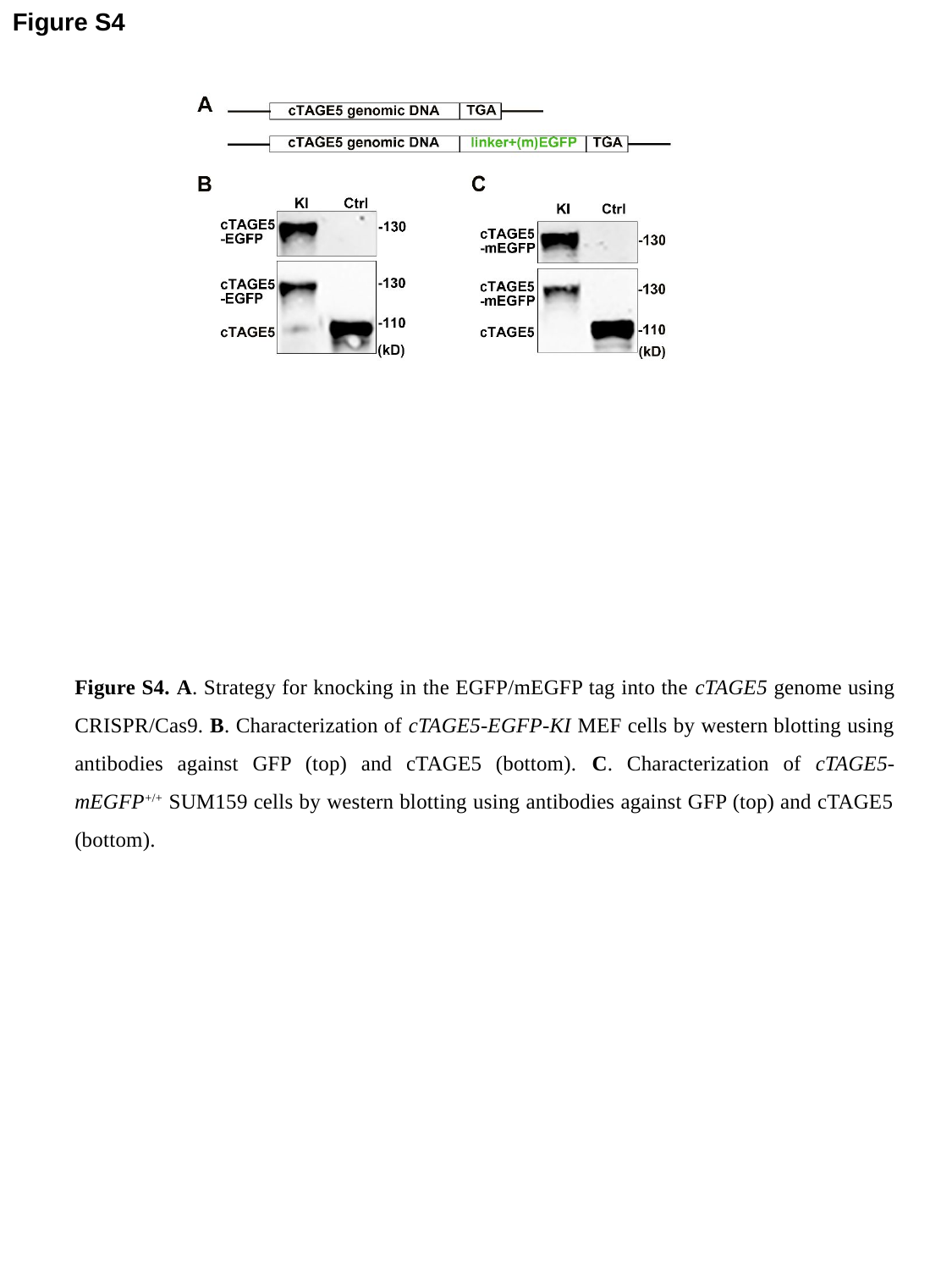

Figure S4
Figure S4. A. Strategy for knocking in the EGFP/mEGFP tag into the cTAGE5 genome using CRISPR/Cas9. B. Characterization of cTAGE5-EGFP-KI MEF cells by western blotting using antibodies against GFP (top) and cTAGE5 (bottom). C. Characterization of cTAGE5-mEGFP+/+ SUM159 cells by western blotting using antibodies against GFP (top) and cTAGE5 (bottom).

## Slide 6
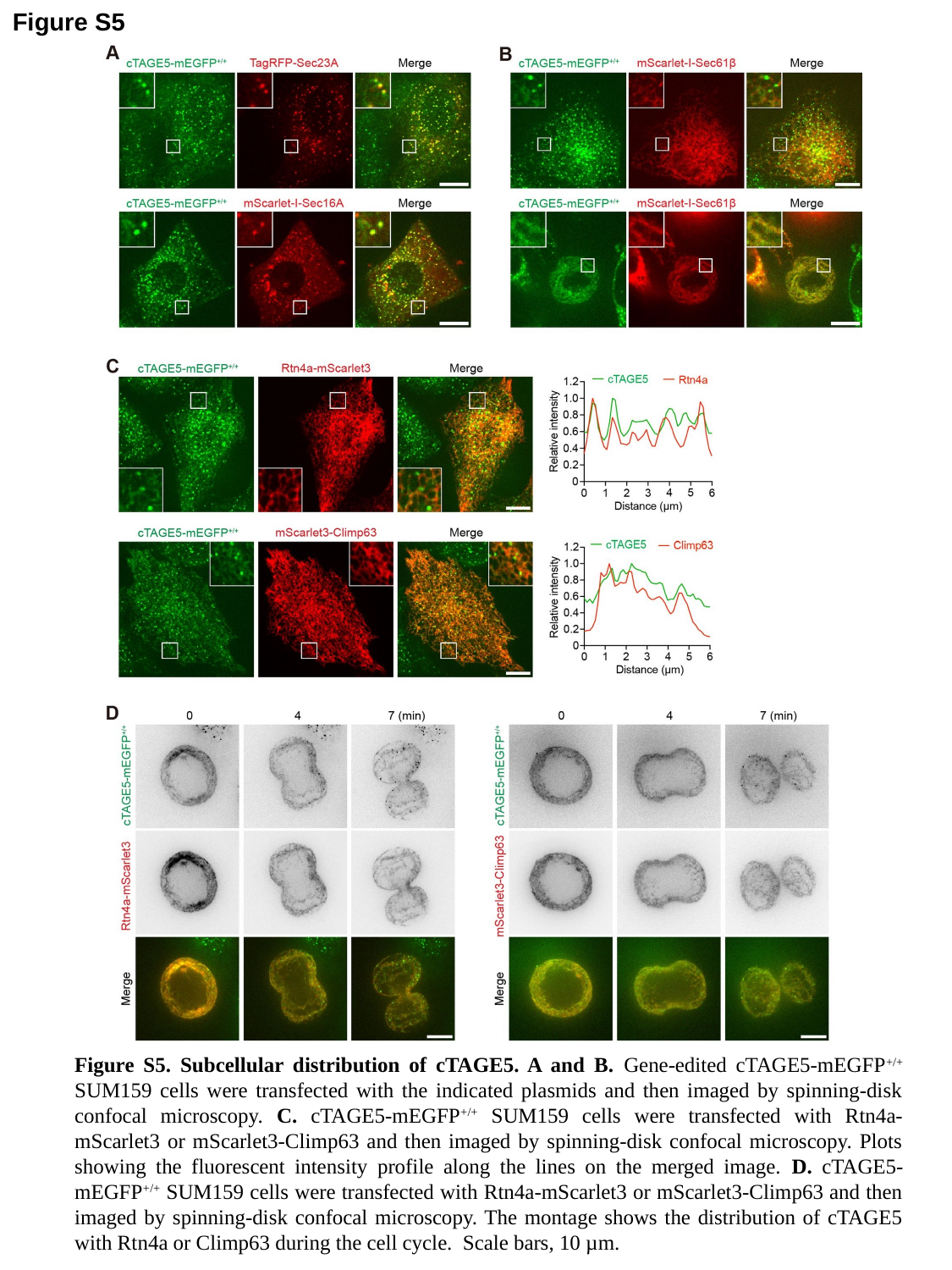

Figure S5
Figure S5. Subcellular distribution of cTAGE5. A and B. Gene-edited cTAGE5-mEGFP+/+ SUM159 cells were transfected with the indicated plasmids and then imaged by spinning-disk confocal microscopy. C. cTAGE5-mEGFP+/+ SUM159 cells were transfected with Rtn4a-mScarlet3 or mScarlet3-Climp63 and then imaged by spinning-disk confocal microscopy. Plots showing the fluorescent intensity profile along the lines on the merged image. D. cTAGE5-mEGFP+/+ SUM159 cells were transfected with Rtn4a-mScarlet3 or mScarlet3-Climp63 and then imaged by spinning-disk confocal microscopy. The montage shows the distribution of cTAGE5 with Rtn4a or Climp63 during the cell cycle. Scale bars, 10 µm.

## Slide 7
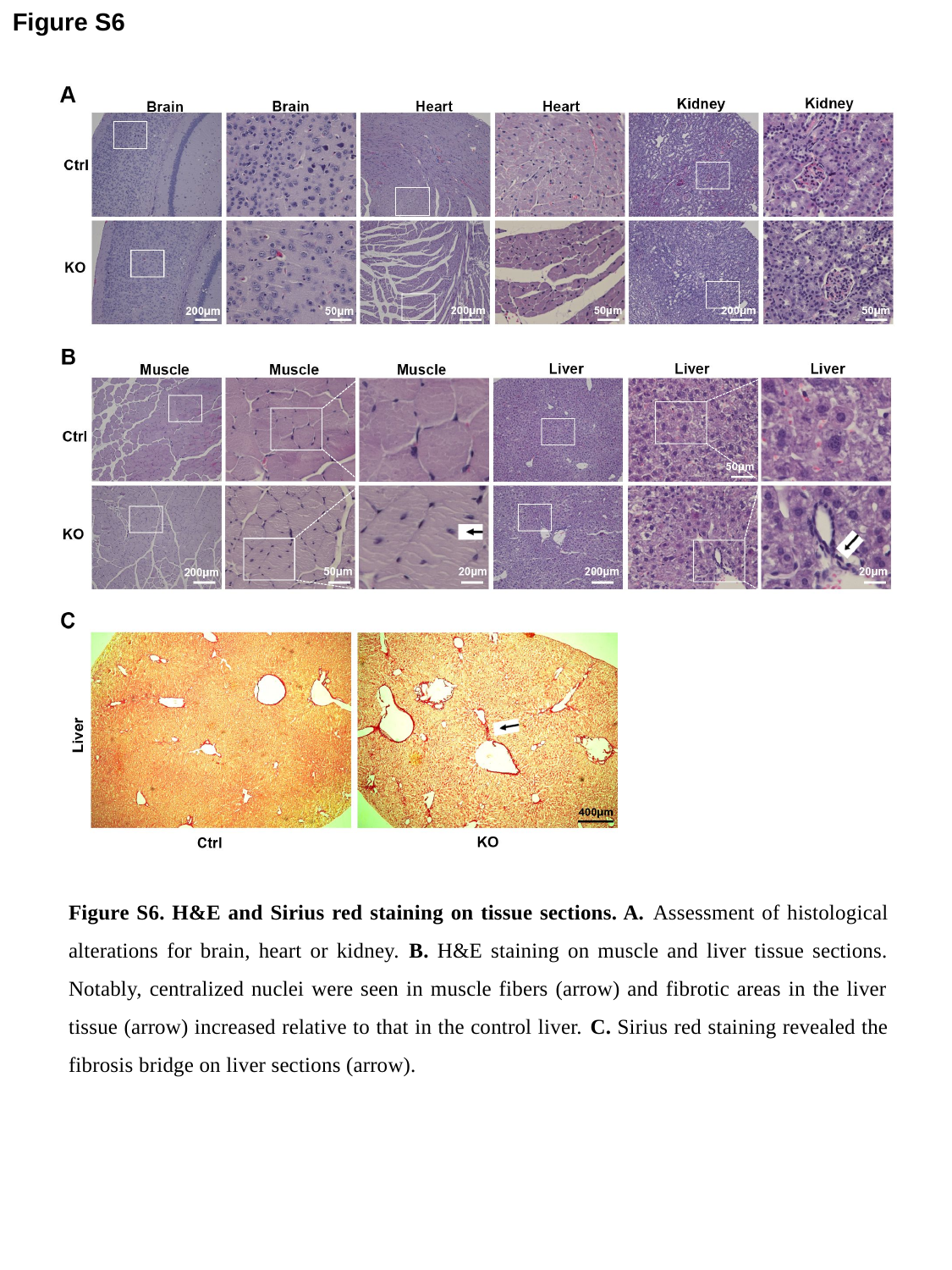

Figure S6
Figure S6. H&E and Sirius red staining on tissue sections. A. Assessment of histological alterations for brain, heart or kidney. B. H&E staining on muscle and liver tissue sections. Notably, centralized nuclei were seen in muscle fibers (arrow) and fibrotic areas in the liver tissue (arrow) increased relative to that in the control liver. C. Sirius red staining revealed the fibrosis bridge on liver sections (arrow).

## Slide 8
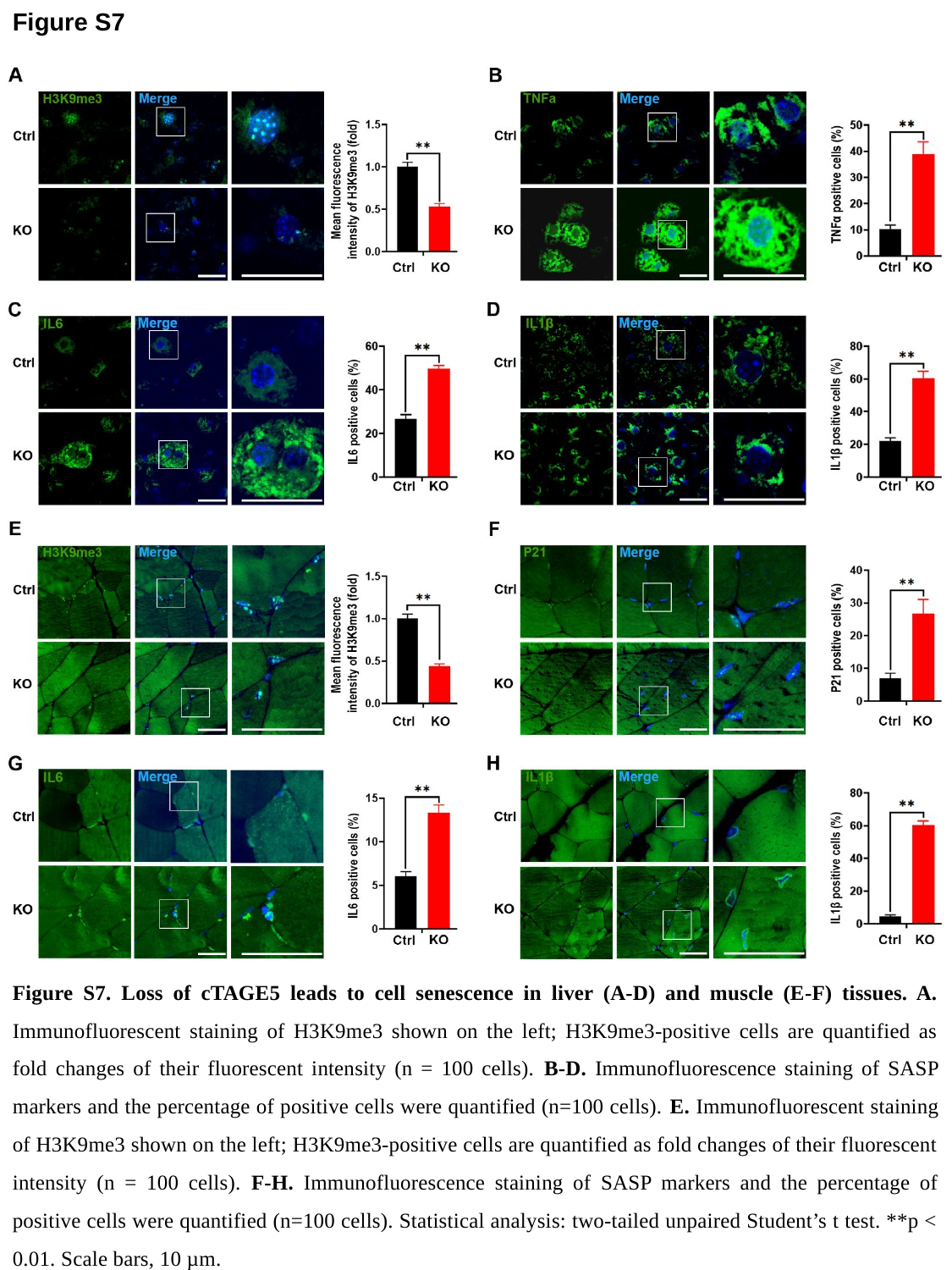

Figure S7
Figure S7. Loss of cTAGE5 leads to cell senescence in liver (A-D) and muscle (E-F) tissues. A. Immunofluorescent staining of H3K9me3 shown on the left; H3K9me3-positive cells are quantified as fold changes of their fluorescent intensity (n = 100 cells). B-D. Immunofluorescence staining of SASP markers and the percentage of positive cells were quantified (n=100 cells). E. Immunofluorescent staining of H3K9me3 shown on the left; H3K9me3-positive cells are quantified as fold changes of their fluorescent intensity (n = 100 cells). F-H. Immunofluorescence staining of SASP markers and the percentage of positive cells were quantified (n=100 cells). Statistical analysis: two-tailed unpaired Student’s t test. **p < 0.01. Scale bars, 10 µm.

## Slide 9
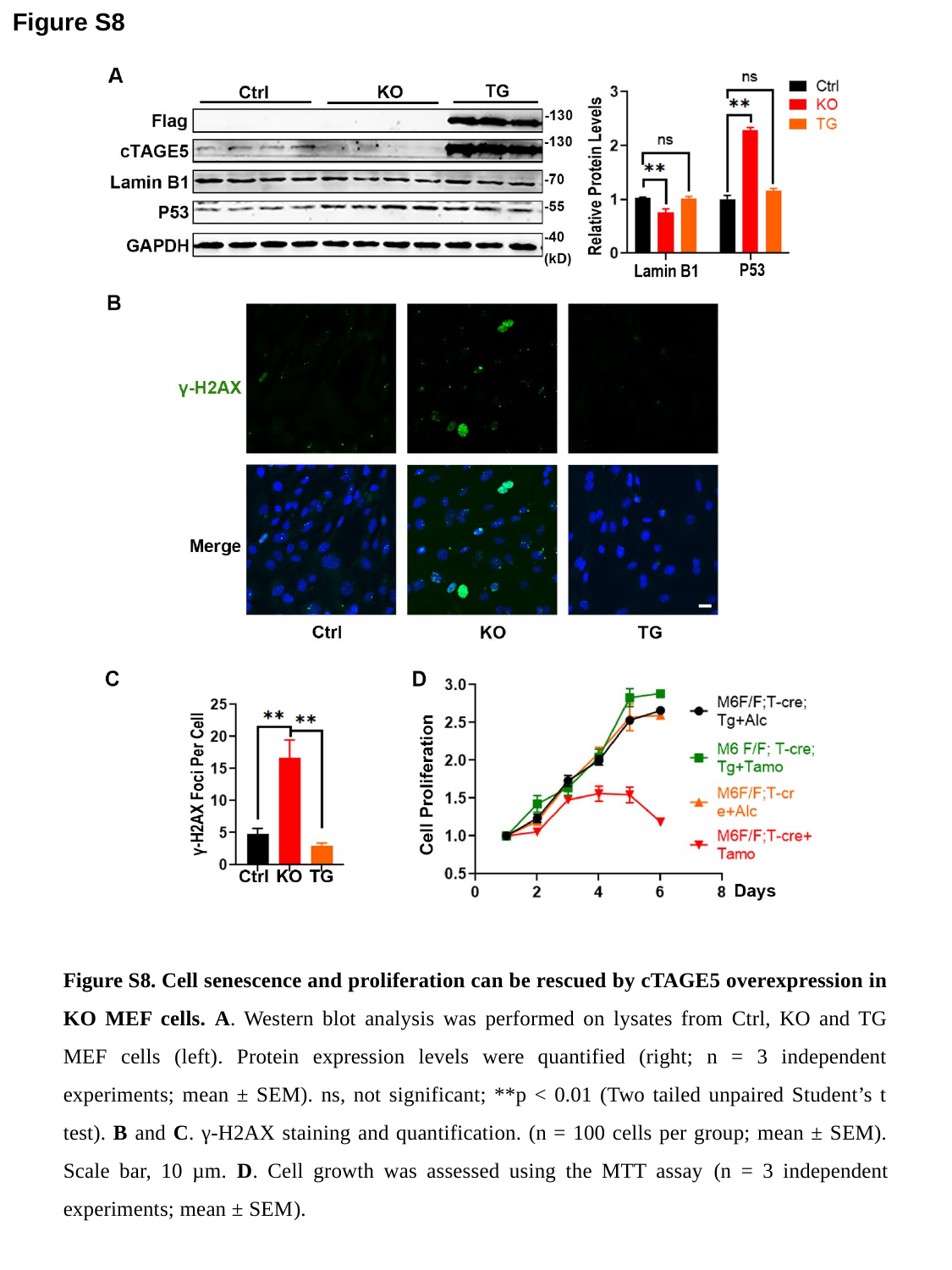

Figure S8
Figure S8. Cell senescence and proliferation can be rescued by cTAGE5 overexpression in KO MEF cells. A. Western blot analysis was performed on lysates from Ctrl, KO and TG MEF cells (left). Protein expression levels were quantified (right; n = 3 independent experiments; mean ± SEM). ns, not significant; **p < 0.01 (Two tailed unpaired Student’s t test). B and C. γ-H2AX staining and quantification. (n = 100 cells per group; mean ± SEM). Scale bar, 10 µm. D. Cell growth was assessed using the MTT assay (n = 3 independent experiments; mean ± SEM).
